# Aloe‐Emodin Targeting FOXC2 Disrupts NETs Formation and EMT‐Driven Postoperative Peritoneal Adhesion Through TGF‐β1‐Smad2/3 Pathway

**DOI:** 10.1002/advs.202511013

**Published:** 2025-10-13

**Authors:** Lili Yang, Yunda Fang, Yuheng Lian, Ziyang Kong, Jia Miao, Yanqi Chen, Wen Li, Feiyan Chen, Bin Zhang, Yao Chen, Yaoyao Bian

**Affiliations:** ^1^ Jiangsu Provincial Engineering Research Center of TCM External Medication Development and Application Nanjing University of Chinese Medicine Nanjing 210023 China; ^2^ Jingwen Library Nanjing University of Chinese Medicine Nanjing 210023 China; ^3^ School of First Clinical Medicine Nanjing University of Chinese Medicine Nanjing 210023 China; ^4^ Faculty of Chinese Medicine Macau University of Science and Technology Taipa Macau 999078 China; ^5^ School of Health Preservation and Rehabilitation Nanjing University of Chinese Medicine Nanjing 210023 China; ^6^ Research and Innovation Center College of Traditional Chinese Medicine Integrated Chinese and Western Medicine College Nanjing University of Chinese Medicine Nanjing 210023 China; ^7^ Department of Gastroenterology Ningbo Municipal Hospital of TCM Affiliated Hospital of Zhejiang Chinese Medical University Ningbo 315012 China; ^8^ School of Pharmacy Nanjing University of Chinese Medicine Nanjing 210023 China; ^9^ TCM Rehabilitation Center Jiangsu Second Chinese Medicine Hospital Nanjing 210023 China

**Keywords:** aloe‐emodin, FOXC2, neutrophil extracellular traps, peritoneal fibrosis, postoperative peritoneal adhesion

## Abstract

Postoperative peritoneal adhesion (PPA) develops through TGF‐β1‐driven fibrotic remodeling, characterized by neutrophil extracellular trap (NETs)‐induced aberrant epithelial‐to‐mesenchymal transition (EMT) deposition. Although aloe‐emodin (AE) exhibits anti‐fibrosis potential, its molecular mechanisms remain elusive. Forkhead box protein C2 (FOXC2) is a critical regulator of fibrotic tissue formation, yet its role in PPA is unknown. Here, it is demonstrated that FOXC2 expression is elevated in human ileostomy tissue, PPA rodent model, and TGF‐β1‐exposed peritoneal mesothelial cells (PMCs), where it orchestrates NETs formation and extracellular matrix (ECM) remodeling. Mechanically, CRISPR/Cas‐based knockdown and overexpression of FOXC2 alter EMT changes in PMCs, which is achieved via TGF‐β1‐Smad2/3 signaling. FOXC2 functions as a dual mediator and amplifier through the TGF‐β1‐Smad2/3 pathway feedback loop to drive EMT alterations. Its overexpression further induces neutrophil recruitment and NETs formation, exacerbating EMT in PMCs. Notably, AE ameliorates FOXC2‐driven peritoneal fibrosis by impeding NETs formation and EMT changes through the TGF‐β1‐Smad2/3 pathway. Moreover, AE binds directly to FOXC2, and the Ser125 residue is critical for the binding of FOXC2 to AE. These findings identify FOXC2 as a pivotal effector in fibrotic responses during PPA formation and reveal that AE targeting the Ser125 residue of FOXC2 may be a promising therapeutic approach to attenuate PPA.

## Graphical abstract

Forkhead box protein C2 (FOXC2) is highlighted as a pivotal effector in postoperative peritoneal adhesion (PPA) formation. Mechanically, FOXC2 mediates NETs‐induced EMT alterations through the TGF‐β1‐Smad2/3 pathway. Aloe‐emodin (AE) ameliorates FOXC2‐driven fibrosis by blocking NETs formation and EMT changes through this pathway. AE binds FOXC2 at Ser125, establishing FOXC2 as a therapeutic target and demonstrating AE's potential in attenuating PPA.

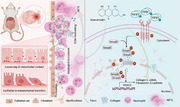

## 1 Introduction

Postoperative peritoneal adhesion (PPA) refers to the formation of irreversible fibrous hyperplasia between the wound site and adjacent organs or the abdominal wall after abdominal surgery. Despite the remarkable advancements in surgical techniques and equipment, PPA remains the most prevalent complication following intra‐abdominal surgeries, with an incidence rate reported to be as high as 90%^[^ ^1^ ^]^ and a recurrence rate reaching 80% among patients who underwent abdominal surgery.^[^ ^2^ ^]^ PPA commonly results in a variety of adverse events, including persistent pain, infertility in females, bowel obstruction, and the development of fistulas.^[^ ^3^ ^]^ A Scottish survey indicates that ≈35% of patients required re‐admission to the hospital due to complications that were either directly or indirectly linked to adhesions.^[^ ^4^, ^5^ ^]^ According to reports, the annual expenses for adhesiolysis, a prevalent surgical treatment for PPA in the United States, amounted to a staggering $2.1 billion.^[^ ^6^ ^]^ Meanwhile, the hospitalization costs associated with small bowel obstruction due to adhesions have reached as high as €16305 in the Netherlands.^[^ ^7^ ^]^ Moreover, a recent systematic review conducted with 22 studies showed that the mortality rate of adhesiolysis among octogenarians is as high as 6–15%.^[^ ^8^ ^]^ The expenses incurred from a second surgery and adhesion‐related complications impose a significant economic burden not only on individuals and families but also on the broader public health system. Therefore, it is urgent to find more effective and novel strategies to prevent PPA.

PPA formation is a dynamic and multi‐stage process involving inflammation, fibrinolysis, angiogenesis, and extracellular matrix (ECM) remodeling.^[^ ^9^ ^]^ When tissue is injured, the coagulation cascade is rapidly activated, and fibrin is produced to form a temporary matrix in response to bleeding and wounds. Subsequently, a series of inflammatory mediators, i.e., cytokines, chemokines, and growth factors, are released, facilitating the recruitment of immune cells, i.e., neutrophils, to the injured sites.^[^ ^10^ ^]^ A variety of stimuli induce neutrophil activation, leading to chromatin decondensation, as well as the release and expulsion of granular proteins, ultimately resulting in the formation of neutrophil extracellular traps (NETs). NETs, in turn, trigger the pro‐inflammatory cascade that further exacerbates the fibrin deposition and peritoneal adhesion.^[^ ^11^ ^]^ Recent studies have indicated that NETs function as important scaffolds for adhesion formation.^[^ ^12^, ^13^ ^]^ NETs can also facilitate epithelial cells through epithelial‐to‐mesenchymal transition (EMT).^[^ ^14^ ^]^ As the first physical barrier after tissue injury, uncontrolled proliferation of peritoneal mesothelial cells (PMCs), a type of simple squamous epithelial cell, undergoes the EMT, which further drives the fibrotic progression. This suggests that EMT induced by NETs formation may play a pivotal role in the PPA occurrence and development.^[^ ^12^ ^]^ As a principal fibrotic mediator of EMT, transforming growth factor‐β1 (TGF‐β1) also plays an indispensable role in PMC‐induced fibrosis and PPA formation.^[^ ^15^ ^]^ It can bind with specific receptors on the cell membrane, and then the receptors are activated and phosphorylated by the downstream Smad 2 and/or Smad3, which then form heterotrimeric complexes with Smad4. These complexes translocate to the nucleus, where they bind to specific DNA sequences of fibrotic or ECM‐associated target genes and govern their transcription and expression, ultimately contributing to the formation of PPA.^[^ ^16^ ^]^ Our previous study also revealed the crucial significance of the TGF‐β1‐Smad2/3 signaling pathway in the development of peritoneal adhesions.^[^ ^17^ ^]^ Moreover, Forkhead box protein C2 (FOXC2), as a key transcription regulator, is induced when epithelial cells undergo EMT mediated by TGF‐β.^[^ ^18^ ^]^ In turn, FOXC2 interacts with Smads involved in the TGF‐β signaling pathway to induce EMT, either by repressing epithelial genes or activating mesenchymal genes.^[^ ^19^ ^]^ The above evidence implies that FOXC2 might be involved in the TGF‐β‐Smads signal cascade and EMT formation. Nevertheless, the underlying mechanism by which FOXC2 contributes to NETs formation and subsequent EMT through the TGF‐β/Smad pathway in the pathogenesis of peritoneal adhesion formation remains largely unknown.

Our previous studies have found that *Huoxuetongfu* Formula (HXTF) has potential effects in preventing PPA formation and development.^[^ ^20^, ^21^ ^]^ Aloe‐emodin (AE, 1,8‐dihydroxy‐3‐hydroxymethyl‐anthraquinone), a natural anthraquinone derivative, is derived from the herbal medicine *Dahuang* (*Rheum palmatum L*.), which is one of the main active constituents of HXTF. AE is attracting growing interest because of its multifaceted pharmacological properties, such as anti‐inflammatory, anti‐fibrosis, anti‐oxidant, and immunomodulatory effects.^[^ ^22^ ^]^ Recent studies have shown that AE was predicted to possess plasma stability with a half‐life of 68.11 h and a plasma protein binding rate of 42.67%, indicating that AE could be considered a promising drug candidate.^[^ ^23^, ^24^ ^]^ However, the potential activity of AE to alleviate PPA formation has not been reported yet. Currently, we aim to explore the critical role of FOXC2 in peritoneal adhesion formation and the underlying mechanism by which FOXC2 mediates NETs formation to the EMT deposition and its role in the TGF‐β1‐Smads pathway. We also sought to evaluate the anti‐adhesive effects of AE targeting FOXC2 on PPA formation. Our discoveries demonstrated that FOXC2 may serve as a key fibrotic regulator in PPA formation, and AE targeting FOXC2 may be a promising therapeutic strategy for PPA.

## 2 Results

### 2.1 FOXC2 is Elevated in Human Ileostomy Tissue, PPA Model, and TGF‐β1‐Exposed PMCs

**Figure 1:**
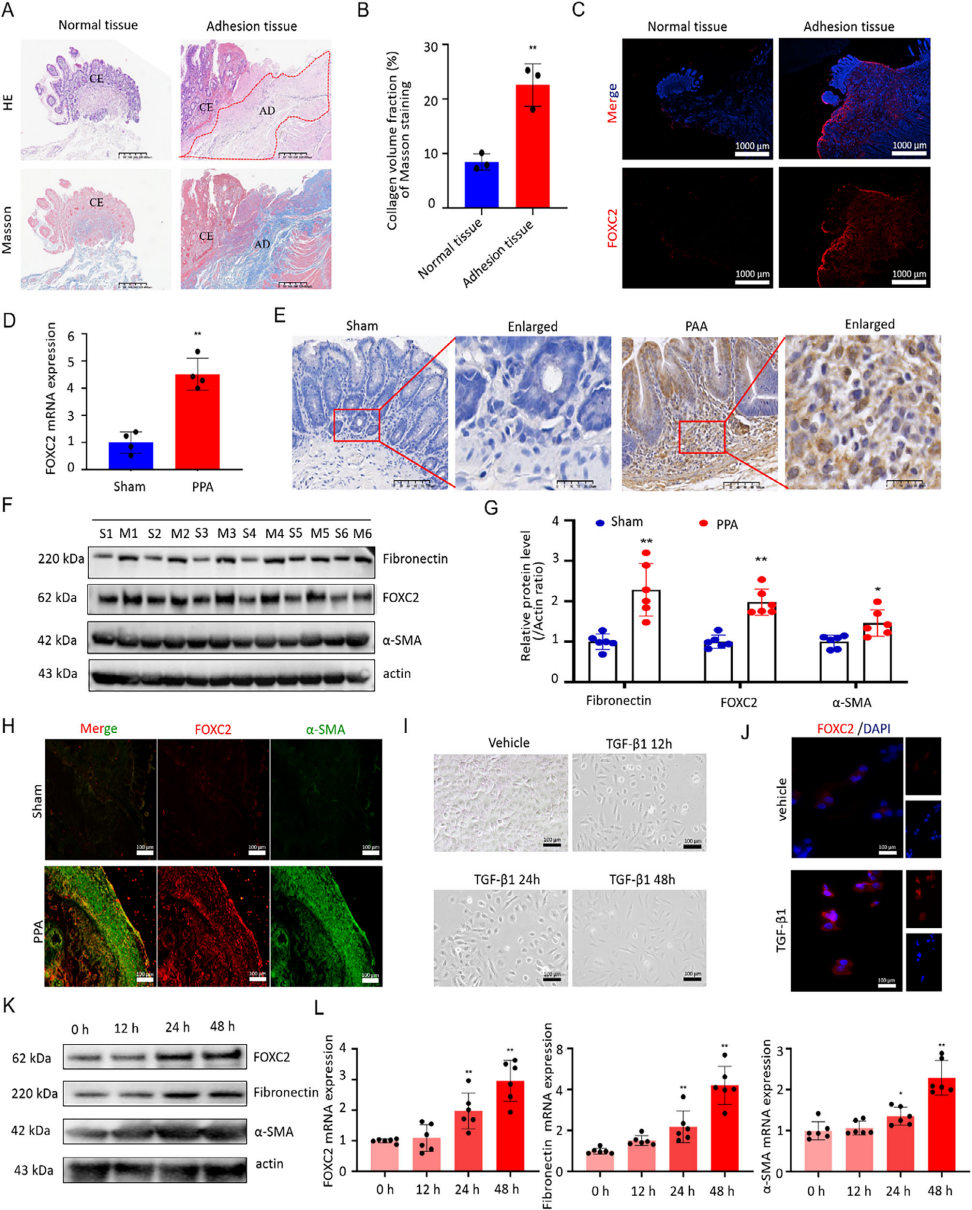
FOXC2 is elevated in human ileostomy tissue, PPA model, and TGF‐β1‐exposed PMCs. A) H&E and Masson staining of human ileostomy tissue. CE: cecum, AD: adhesion. Scale bar = 400 µm. B) Collagen volume fraction in Masson‐stained cecum sections of human ileostomy tissue. n = 3 per group. Compared with the normal tissue, ^**^ *p* < 0.01. C) Immunofluorescence staining images of FOXC2 (red) in cecum sections of human ileostomy tissue. Scale bar = 1000 µm. D) qRT‐PCR analysis of FOXC2 mRNA expression in cecum sections of the PPA model. n = 4 per group. Compared with the sham group, ^**^ *p* < 0.01. E) Immunohistochemical images of FOXC2 of cecum sections in the PPA model. Scale bar = 100 µm in unenlarged images. Scale bar = 25 µm in enlarged images. F,G) Western blot analyses of FOXC2 and ECM‐related proteins (Fibronectin and α‐SMA) levels of cecum sections in the PPA model. n = 6 per group (S1‐S6, sham groups; M1‐M6, PPA model groups). Compared with the sham group, ^*^ *p* < 0.05, ^**^ *p* < 0.01. H) Double‐immunofluorescence staining images of FOXC2 (red) and α‐SMA (green) of cecum sections in the PPA model. Scale bar = 100 µm. I) Morphology of PMCs treated with 10 ng mL^−1^ TGF‐β1 at different exposure time points. Scale bar = 100 µm. J) Immunofluorescence staining images of FOXC2 (red) in PMCs treated with 10 ng mL^−1^ TGF‐β1 for 24 h. Scale bar = 100 µm. K) Western blot analyses of FOXC2 and ECM‐related proteins (Fibronectin and α‐SMA) expressions in PMCs treated with 10 ng mL^−1^ TGF‐β1 at different exposure time points. L) qRT‐PCR analysis of mRNA expressions of FOXC2 and ECM‐related markers (Fibronectin and α‐SMA) in PMCs treated with 10 ng mL^−1^ TGF‐β1 at different exposure time points. n = 6 per group. Compared with the control, ^*^ *p* < 0.05, ^**^ *p* < 0.01.

Human ileostomy tissues were retrieved for hematoxylin and eosin (H&E) and Masson staining. It showed that massive inflammatory cell infiltration and dense collagen fiber bands were deposited in adhesive tissues when compared with the normal tissue, as displayed in **Figure** **1**A. Similar results could be found in the semi‐quantitative assay of Masson staining, as presented in Figure 1B. The analysis revealed a significantly higher collagen volume fraction in the human adhesion tissues (22.6 ± 3.9%) compared to the normal tissues (8.5 ± 1.5%, *p* <0.01). Immunofluorescent assay showed that FOXC2 was highly expressed in human adhesion tissues, as shown in Figure 1C. To assess the expression of FOXC2 in the PPA rodent model at molecular level, we then conducted the qRT‐PCR analysis and found that the expression levels of FOXC2 were dramatically increased in the PPA group (3.5 ± 0.6) in comparison with the sham group (1.0 ± 0.4, *p* < 0.01; Figure 1D). The results of immunohistochemical staining indicated that the staining intensity in the PPA group was stronger, with clearly distinguishable cytoplasm and deeply stained cytoplasm (Figure 1E). In addition, western blot analysis revealed that the protein expression of FOXC2 (2.0‐fold, *p* <0.01) was elevated in the PPA group, accompanied by the accumulation of ECM‐related proteins, i.e., Fibronectin (2.3‐fold, *p* < 0.01) and α‐smooth muscle actin (α‐SMA; 1.5‐fold, *p* <0.05), when compared with the sham group (Figure 1F,G). The double immunofluorescent analysis revealed that the fluorescence intensities of FOXC2 and α‐SMA in the PPA group were significantly higher than those in the sham group (Figure 1H).

Mesothelial cells, as primary barriers, play an important role in maintaining the stability of the abdominal environment and preventing the formation of adhesions.^[^ ^25^ ^]^ When PMCs were injured by mechanical trauma, they further underwent transdifferentiation, resulting in a substantial contribution to the population of cells displaying a fibrotic phenotype.^[^ ^26^ ^]^ TGF‐β1 is a well‐recognized major regulator of tissue fibrosis, which contributes to the pathogenesis of PPA.^[^ ^27^ ^]^ Herein, we established an in vitro model using TGF‐β1‐stimulated PMCs to further investigate the critical roles of FOXC2 in fibrotic responses. With the extension of exposure time of 10 ng mL^−1^ TGF‐β1, the morphology of PMCs changes from their original cobblestone‐like appearance to elongated or spindle‐shaped forms (Figure 1I). The results of immunofluorescent staining showed that the intensity of FOXC2 was significantly increased in the TGF‐β1‐exposed group in comparison with the vehicle group (Figure 1J). The levels of FOXC2 and ECM‐related proteins (Fibronectin and α‐SMA) were also elevated when exposed to 10 ng mL^−1^ TGF‐β1 at different time points (Figure 1K). Similarly, the results of qRT‐PCR indicated that with prolonged exposure to TGF‐β1, the expression levels of FOXC2, Fibronectin, and α‐SMA increased dramatically (Figure 1L). Collectively, these data suggested that the elevation of FOXC2 might be involved in peritoneal adhesion formation.

### 2.2 FOXC2 is Involved in NETs Formation and ECM Remodeling, Evidenced by the Global Transcriptomic Changes

**Figure 2:**
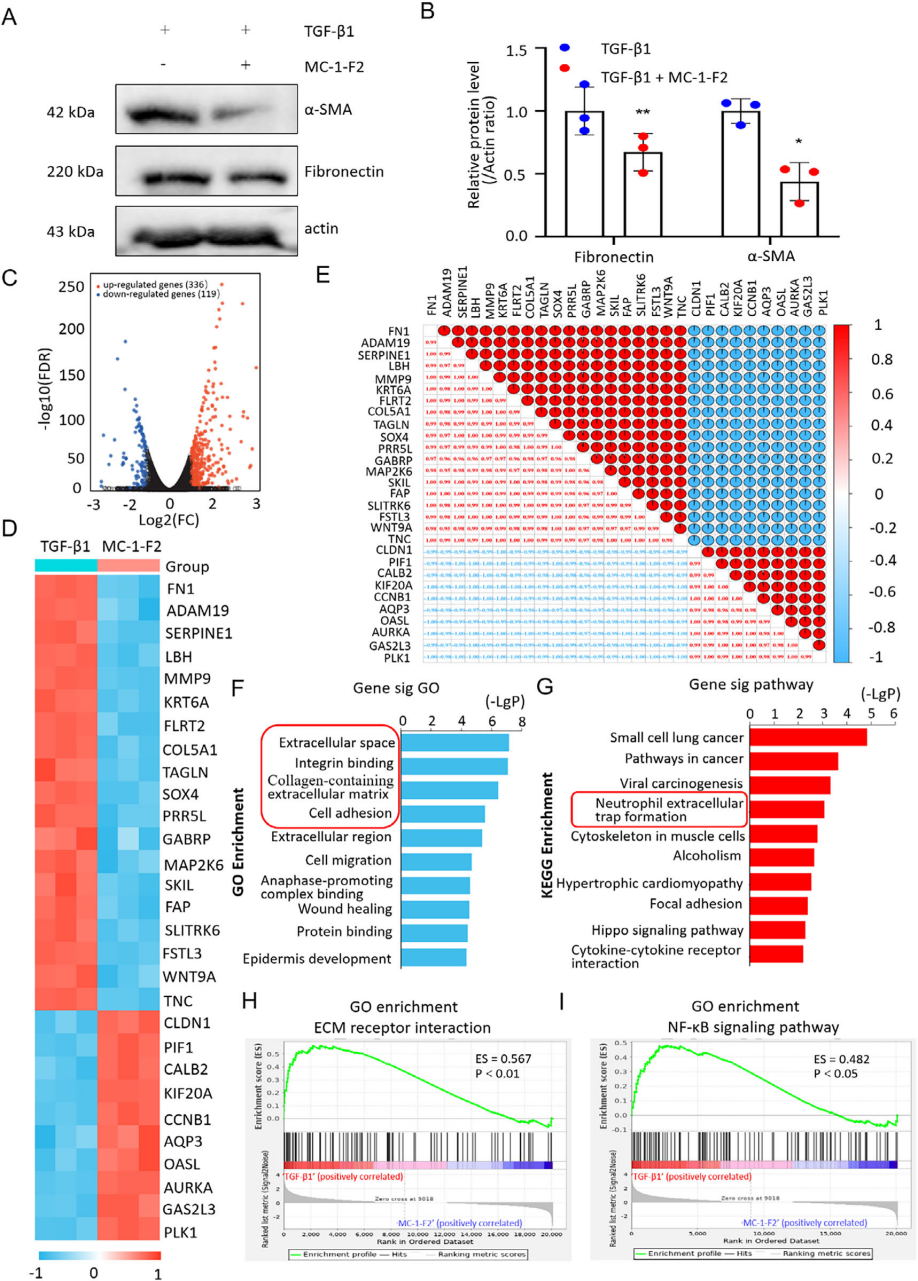
FOXC2 is involved in NETs formation and ECM remodeling. A,B) Western blot analyses of ECM‐related proteins (α‐SMA and Fibronectin) levels in 10 ng mL^−1^ TGF‐β1 pre‐treated PMCs with or without MC‐1‐F2 treatment. n = 3 per group. Compared with the TGF‐β1 group, ^*^ *p* < 0.05, ^**^ *p* < 0.01. C) Volcano plot of 10 ng mL^−1^ TGF‐β1 pre‐treated PMCs with or without MC‐1‐F2. The red dots represented up‐regulated RNAs, while the blue dots represented down‐regulated RNAs. D) Heat map indicated differentially expressed genes in each group. E) Correlation heat map indicated the correlation between differentially expressed genes. Red and blue represented positive correlation and negative correlation, respectively. F) GO annotation of differentially expressed genes (top 10). G) KEGG enrichment of significantly changed genes (top 10). H,I) GSEA plots for the gene enrichment with differentially expressed genes based on the GO database.

MC‐1‐F2, an inhibitor of FOXC2, has been demonstrated to induce 26S proteasome‐mediated degradation of FOXC2. This degradation process may subsequently lead to the inhibition of the transcriptional regulation of FOXC2.^[^ ^28^ ^]^ PMCs stimulated by 10 ng mL^−1^ TGF‐β1 for 24 h were then treated with or without 20 µM MC‐1‐F2 for another 24 h. The results of western blot analysis indicated that the levels of ECM‐related proteins (α‐SMA; 0.4‐fold, *p* < 0.01, and Fibronectin; 0.7‐fold, *p* < 0.05) were down‐regulated in the MC‐1‐F2‐treated group when compared with the untreated group (**Figure** **2**A,B). To further explore the potential targets regulated by FOXC2 in PMCs, the above cells were subjected to RNA sequencing. The transcriptomic results showed that the abundance of 336 mRNAs increased, while the abundance of 119 mRNAs decreased, as a result of FOXC2 inhibition (Figure 2C). In comparison with the TGF‐β1 group, the mRNA expressions of FN1 (a Fibronectin subtype) and COL5A1 (a collagen‐encoding gene) were significantly decreased while the mRNA expression of CLDN1 (Claudin‐1, an intracellular tight‐junction marker) was markedly increased (Figure 2D). The RNA levels of FN1 were positively correlated with COL5A1, while negatively correlated with CLDN1 (Figure 2E). Gene Ontology (GO) annotation indicated that significantly expressed genes were annotated in extracellular space, integrin binding, collagen‐containing extracellular matrix, and cell adhesion (Figure 2F). Kyoto Encyclopedia of Genes and Genomes (KEGG) enrichment revealed that the differentially expressed genes were enriched in neutrophil extracellular trap formation and Hippo signaling pathway, as displayed in Figure 2G. Gene Set Enrichment Analysis (GSEA) based on the GO database indicated that the significantly expressed genes were mainly associated with ECM receptor interaction, and NF‐κB signaling pathway (Figure 2H,I). Recent evidence suggested that NETs serve as important scaffolds for adhesion formation.^[^ ^12^, ^13^ ^]^ NETs can promote ECM remodeling by releasing various pro‐inflammatory cytokines, chemokines, and growth factors that are involved in EMT induction. Additionally, NETs can directly interact with ECM components, i.e., collagen and fibronectin, facilitating ECM remodeling and deposition. Herein, NETs formation was selected for analysis. Taken together, the results revealed that FOXC2 might be involved in NETs formation and ECM remodeling.

### 2.3 CRISPR/Cas‐Based Knockdown and Overexpression of FOXC2 Alter EMT Changes in PMCs

**Figure 3:**
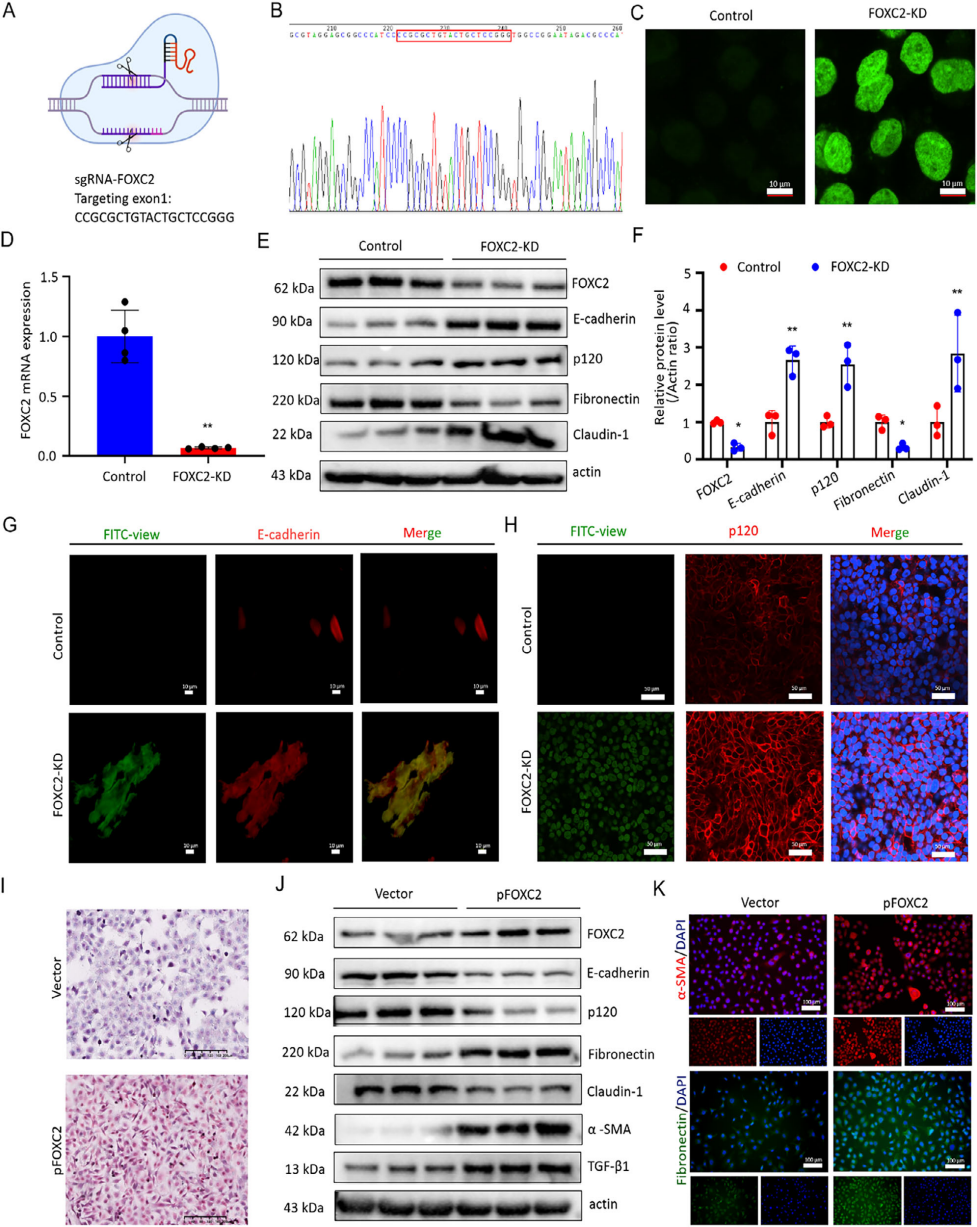
CRISPR/Cas‐based knockdown and overexpression of FOXC2 alter EMT changes in vitro. A) Schematic diagram of the protocol for knocking down FOXC2 by the CRISPR/Cas9 system. B) Sanger sequencing validation of FOXC2 knocked down. C) Immunofluorescence staining images of PMCs with or without FOXC2 knockdown. FOXC2‐knockdown PMCs exhibit green fluorescence, whereas the controls do not display any fluorescence. Scale bar = 10 µm. D) qRT‐PCR analysis of FOXC2 mRNA expression in PMCs with or without FOXC2 knockdown. n = 4 per group. Compared with the control group, ^**^ *p* < 0.01. E,F) Western blot analyses of FOXC2, mesenchymal‐related protein (Fibronectin), and mesothelial‐related proteins (E‐cadherin, p120, Claudin‐1) levels in PMCs with or without FOXC2 knockdown. n = 3 per group. Compared with the control group, ^**^ *p* < 0.01. ^*^ *p* < 0.05. G) Immunofluorescence staining images of E‐cadherin (red) in PMCs with or without FOXC2 knockdown. Scale bar = 10 µm. H) Immunofluorescence staining images of p120 (red) and DAPI (blue) in PMCs with or without FOXC2 knockdown. Scale bar = 50 µm. I) Direct red 80 of PMCs transfected with empty or pFOXC2 vector for 24 h. Scale bar = 200 µm. J) Western blot analyses the abundance of FOXC2, TGF‐β1, mesenchymal‐related proteins (Fibronectin, α‐SMA), and mesothelial‐related proteins (E‐cadherin, p120, Claudin‐1) in PMCs transfected with empty or pFOXC2 vector for 24 h. K) Immunofluorescence staining images of α‐SMA (red) and Fibronectin (green) in PMCs transfected with empty or pFOXC2 vector for 24 h, respectively. Scale bar = 100 µm.

To explore the critical roles of FOXC2 in the ECM changes of PMCs. CRISPR/Cas‐based FOXC2 knockdown (FOXC2‐KD) in PMCs was employed (**Figure** **3**A,B), and the FOXC2‐KD PMCs exhibited green fluorescence under a fluorescence microscope (Figure 3C). Compared with the control group, the FOXC2‐KD PMCs showed a 93.2% decrease in mRNA expression (*p* < 0.01, Figure 3D) and a 67.3% reduction in protein level (*p* < 0.05), indicating a high knockdown efficacy (Figure 3E,F). Western blot analysis showed that after FOXC2 knockdown, there was a significant decrease in the protein level of Fibronectin (0.3‐fold, *p* < 0.05) in CRISPR‐treated PMCs compared to those in controls. It is said that E‐cadherin and p120 can form an adhesion complex on the cell membrane to sustain the adhesiveness and polarity of epithelial cells. When this complex is disrupted, epithelial cells undergo EMT changes and transform into cells with mesenchymal characteristics.^[^ ^29^ ^]^ Besides, Claudin‐1, a member of the tight junction protein family, has been reported to directly promote EMT through its interaction with defined EMT‐related transcription factors and signaling pathways.^[^ ^30^ ^]^ Herein, E‐cadherin, p120, and Claudin‐1 were sought to be analyzed. There was a marked increase in the levels of mesothelial‐related proteins (E‐cadherin; 2.7‐fold, *p* < 0.01, p120; 2.7‐fold, *p* < 0.01, and Claudin‐1; 2.8‐fold, *p* < 0.01) in FOXC2‐KD PMCs in comparison with controls, as shown in Figure 3E,F. Immunofluorescent staining also confirmed the augmentation of both E‐cadherin and p120 in the FOXC2‐CRISPR‐treated PMCs as compared with the controls (Figure 3G,H).

In addition, PMCs transfected with either a FOXC2 overexpression vector (pFOXC2) or an empty vector were also generated to investigate the pivotal roles of FOXC2 on the EMT changes in PMCs. Significantly positive staining of direct red 80 was observed with high collagen deposition in PMCs when transfected with pFOXC2 (Figure 3I). Western blot analysis was used to evaluate the transfection efficacy, as presented in Figure 3J. FOXC2 overexpression significantly increased the synthesis of ECM‐related protein, i.e., Fibronectin, and upregulated the protein levels of TGF‐β1. It also upsurged the protein levels of mesenchymal‐related protein (α‐SMA), which was involved in ECM deposition. By contrast, FOXC2 overexpression markedly decreased the abundance of mesothelial‐related proteins, such as E‐cadherin, p120, and Claudin‐1. Immunofluorescence co‐staining additionally confirmed the augmentation of α‐SMA and Fibronectin in PMCs upon FOXC2 overexpression (Figure 3K). Together, these findings revealed that FOXC2 might serve as a crucial determinant for PMCs to undergo EMT changes.

### 2.4 FOXC2 Driving EMT Changes in PMCs may be Achieved Through the TGF‐β1‐Smad2/3 Signaling Feedback Loop

**Figure 4:**
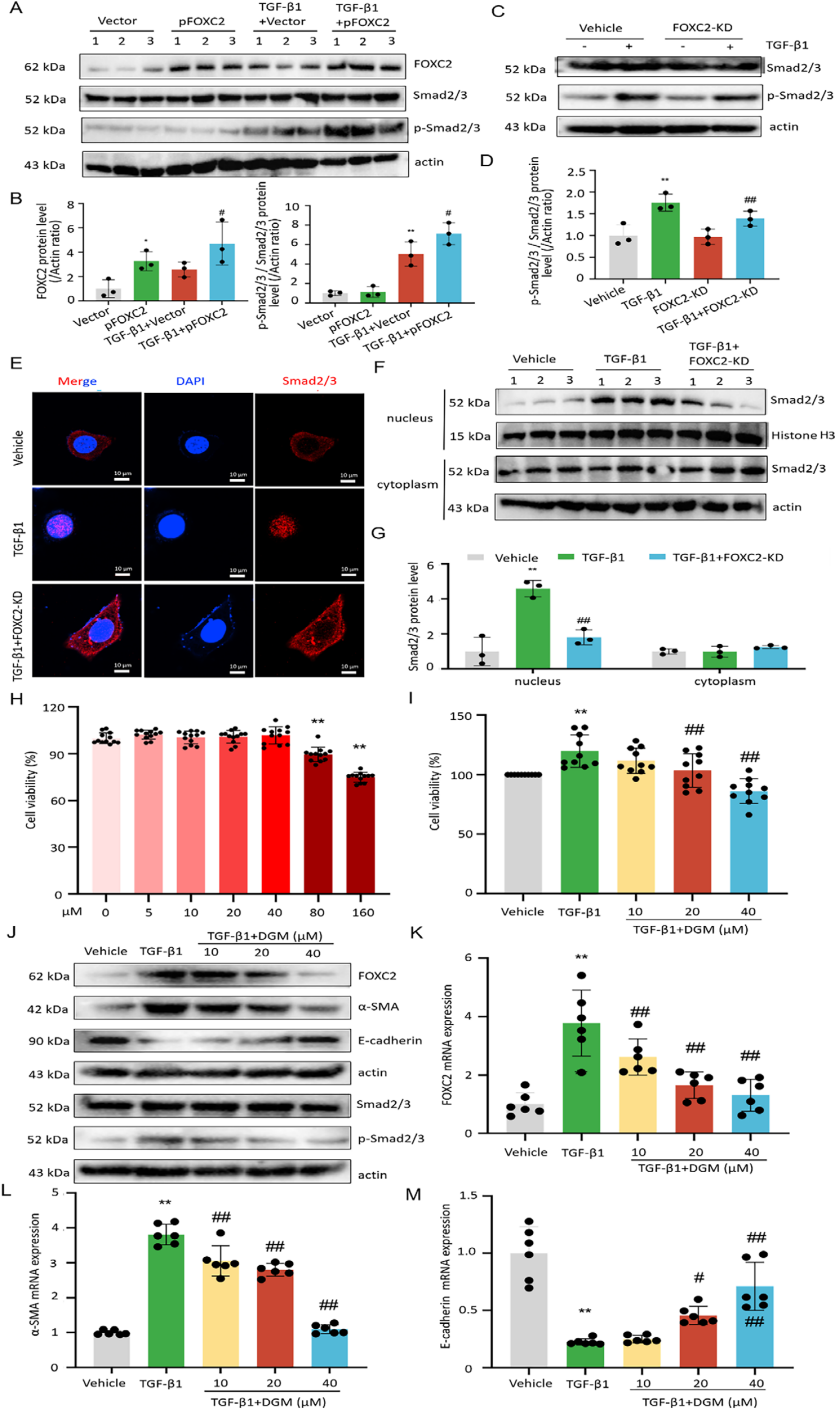
FOXC2 driving EMT changes in PMCs may be achieved through the TGF‐β1‐Smad2/3 signaling feedback loop. A,B) Western blot analyses of FOXC2, Smad2/3, and p‐Smad2/3 protein levels among different groups. n = 3 per group. Compared with the vector group, ^**^ *p* < 0.01, ^*^ *p* < 0.05. Compared with the vector group in the presence of TGF‐β1, ^#^ *p* < 0.05. C,D) Western blot analyses of Smad2/3 and p‐Smad2/3 protein levels among different groups. n = 3 per group. Compared with the vehicle group, ^**^ *p* < 0.01. Compared with the FOXC2‐KD group, ^##^ *p* < 0.01. E) Immunofluorescence staining images of nuclear accumulation of TGF‐β1‐induced Smad2/3 (red) in FOXC2‐KD PMCs. Scale bar = 10 µm. F,G) Western blot analyses of Smad2/3 protein abundance in the cytoplasm and nucleus among different groups. n = 3 per group. Compared with the vehicle group, ^**^ *p* < 0.01. Compared with the TGF‐β1, ^##^ *p* < 0.01. H) The cell viability of PMCs treated with different concentrations of DGM (5, 10, 20, 40, 80, and 160 µM) for 24 h by CCK8 assay. n = 12 per group. Compared with the vehicle group, ^**^ *p* < 0.01. I) The cell viability of PMCs treated with TGF‐β1 and different concentrations of DGM (10, 20, and 40 µM) for 24 h by CCK8 assay. n = 10 per group. Compared with the vehicle group, ^**^ *p* < 0.01. Compared with the TGF‐β1 group, ^##^ *p* < 0.01. J) Western blot analyses of the protein abundance of FOXC2, α‐SMA, E‐cadherin, Smad2/3, and p‐Smad2/3 in PMCs treated with TGF‐β1 and different concentrations of DGM (10, 20, and 40 µM) for 24 h. K–M) qRT‐PCR analysis of mRNA expressions of FOXC2, α‐SMA, and E‐cadherin in PMCs treated with TGF‐β1 and different concentrations of DGM (10, 20, and 40 µM) for 24 h. n = 6 per group. Compared with the vehicle group, ^**^ *p* < 0.01. Compared with the TGF‐β1 group, ^##^ *p* < 0.01, ^#^ *p* < 0.05.

The TGF‐β/Smad pathway is recognized as a key regulatory mechanism in tissue fibrosis development.^[^ ^31^ ^]^ Elevated TGF‐β1 expression in peritoneal tissues demonstrated a positive association with increased adhesion occurrence.^[^ ^32^ ^]^ Smad downstream molecules, which are intracellular proteins, function as critical mediators in the TGF‐β1 signaling cascade, enabling signal transduction from the cell membrane to the nucleus and subsequently stimulating target gene transcription.^[^ ^33^ ^]^ Notably, FOXC2‐Smad complexes can orchestrate the EMT program through coordinated dual regulation of mesothelial gene repression and mesenchymal gene activation.^[^ ^19^ ^]^ We further hypothesize the regulatory mechanism between FOXC2 and the TGF‐β1‐Smad pathway. Herein, we explored the effects of CRISPR/Cas‐based knockdown and overexpression of FOXC2 on TGF‐β1‐Smad signaling activity. As displayed in **Figure** **4**A–D, the western blot experiments indicated that overexpression of FOXC2 amplified TGF‐β1‐induced phosphorylation of Smad2/3. Smad2/3 was phosphorylated and activated by TGF‐β1, whereas this process was impeded by FOXC2 knockdown. Immunofluorescent staining showed that upon TGF‐β1 stimulation, Smad2/3 was accumulated in the nuclei of PMCs. However, FOXC2 knockdown largely attenuated the TGF‐β1‐induced Smad2/3 nuclear accumulation (Figure 4E). Additionally, the distributions of Smad2/3 in the cytoplasm and nucleus were detected by western blot assay, respectively. It suggested that the nuclear localization of Smad2/3 in TGF‐β1‐induced PMCs was impeded by FOXC2 knockdown (Figure 4F,G). Meanwhile, a TGF‐β1‐Smad inhibitor 3‐cyclopropylmethoxy‐4‐(difluoromethoxy) benzoic acid (DGM) was employed to identify the specific role of the TGF‐β1‐Smad2/3 signaling axis in the regulation of FOXC2 expression. As shown in Figure 4H, no toxic effect was observed on PMCs after 24 h of treatment with different concentrations of DGM within 40 µM. When PMCs were treated with TGF‐β1 for 24 h in combination with DGM, we found that DGM could effectively inhibit the cell viability induced by TGF‐β1 (Figure 4I). Based on these findings, non‐toxic concentrations of DGM (10, 20, and 40 µM) were selected for further experiments. As depicted in Figure 4J, FOXC2 protein abundance increased significantly in PMCs after 24 h of TGF‐β1 stimulation. Conversely, treatment with DGM resulted in a marked reduction in FOXC2 protein expression. The results of qRT‐PCR analysis were consistent with those of western blot analysis (Figure 4K). Similarly, TGF‐β1 stimulation for 24 h significantly upregulated α‐SMA protein abundance and downregulated E‐cadherin expression, which were reversed by DGM treatment. The results of western blot analysis were further supported by qRT‐PCR analysis (Figure 4L,M). In addition, the protein expression of Smad2/3 remained largely unchanged, while the protein level of p‐Smad2/3 increased notably under TGF‐β1 stimulation. After DGM treatment, the expression of p‐Smad2/3 decreased markedly, especially at a concentration of 40 µM (Figure 4J). Altogether, the above data implied that FOXC2 driving EMT changes in PMCs was achieved through the TGF‐β1‐Smad2/3 signaling feedback loop.

### 2.5 FOXC2 Induces Neutrophil Recruitment, Facilitates NETs Formation, and Mediates the Following EMT in PMCs

It is said that NETs can trigger the EMT process, thereby enhancing the migration capacity of cancer cells^[^ ^34^ ^]^ and epithelial cells.^[^ ^35^ ^]^ Using a transwell migration assay, neutrophils were co‐cultured with PMCs, which had been pretreated with PMA, pFOXC2 with or without DNase I, respectively (**Figure** **5**A). DAPI staining revealed that FOXC2 overexpression could attract neutrophil migration (Figure 5B). In comparison with the vehicle group, the relative migration of neutrophils exhibited a 3.9‐fold increase in the pFOXC2 group (*p* < 0.01; Figure 5C). It is said that when exposed to specific stimuli, neutrophils are capable of generating NETs. To explore the underlying mechanism of neutrophil recruitment and activation, the expression of citrullinated histone H3 (CitH3; an important component of NETs) was analyzed. Western blot analysis revealed a 2.7‐fold elevation of CiH3 protein levels in the pFOXC2 group when compared with the vehicle group (*p* < 0.01; Figure 5D,E). In the adhesive trapping assay, we observed an extensive web‐like structure of NETs (labeled blue) released from neutrophils stimulated with pFOXC2, which could promote the adhesion of Dil‐labeled wide‐type PMCs (labeled red). When DNase I was used to disrupt the web‐like structure of NETs, the number of adhered PMCs was decreased, demonstrating the NET‐dependent adhesion mechanism (Figure 5F). Notably, NETs derived from pFOXC2‐stimulated neutrophils exhibited significantly reduced adhesion capacity for Dil‐labeled FOXC2‐KD PMCs when compared to wild‐type PMCs (Figure 5G,H). The data indicated that FOXC2 might facilitate neutrophil recruitment to form a web‐like structure of NETs, and FOXC2 in PMCs played a critical role in NETs‐dependent cell adhesion. To further investigate the critical role of FOXC2 in mediating NETs‐induced EMT in PMCs, the expression levels of mesenchymal‐related marker (α‐SMA) and mesothelial‐related marker (E‐cadherin) were analyzed. Western blot analysis revealed an increase in α‐SMA protein abundance and a decrease in E‐cadherin protein abundance in the PMA group compared with the vehicle group. The protein abundance trend in the pFOXC2 group was comparable to that in the PMA group, whereas DNase I treatment reversed this expression pattern (Figure 5I). The results of qRT‐PCR analysis were consistent with those of western blot analysis (Figure 5J). Immunofluorescent staining also confirmed the augmentation of α‐SMA expression and the reduction of E‐cadherin expression in the pFOXC2 group, both of which exhibited similar trends in the PMA group. However, after treatment with DNase I, the immunofluorescent intensities of α‐SMA and E‐cadherin expression in the pFOXC2 group were reversed (Figure 5K). The above discoveries demonstrated that FOXC2 could induce neutrophil recruitment, facilitate NETs formation, and mediate EMT changes.

Figure 5FOXC2 facilitates neutrophil recruitment to form a web‐like structure of NETs and regulates the following EMT changes in vitro. A) Schematic diagram of neutrophil recruitment in a transwell migration assay, where neutrophils were seeded onto the upper chamber, with PMCs co‐cultured onto the lower chamber. B) DAPI staining of PMCs and recruited neutrophils in a 2‐h transwell co‐culture system. White arrows indicated the smaller cell nuclei of transmigrated neutrophils. Scale bar = 50 µm. C) Quantification analysis of transmigrated neutrophils in a 2‐h transwell co‐culture system. n = 3 per group. Compared with the vehicle group, ^**^ *p* < 0.01. D,E) Western blot analyses of CitH3 protein levels in a 4‐h transwell co‐culture system. n = 3 per group. Compared with the vehicle group, ^**^ *p* < 0.01. F) Adhesion assay for Dil‐labeled PMCs trapped within the web‐like structure of NETs when neutrophils were pretreated differently. Scale bar = 10 µm. G) Adhesion assay for Dil‐labeled FOXC2‐KD PMCs trapped within the web‐like structure of NETs when neutrophils were pretreated differently. Scale bar = 10 µm. H) Schematic diagram of adhesion assay of PMCs or FOXC2‐KD PMCs mediated by NETs. I) Western blot analyses of α‐SMA and E‐cadherin protein abundance. J) qRT‐PCR analysis of α‐SMA and E‐cadherin mRNA expressions. n = 6 per group. Compared with the vehicle group, ^**^ *p* < 0.01. Compared with the PMA group, ^##^ *p* < 0.01. K) Immunofluorescence staining images of α‐SMA and E‐cadherin expressions (stained with red). Scale bar = 10 µm.

**Figure:**
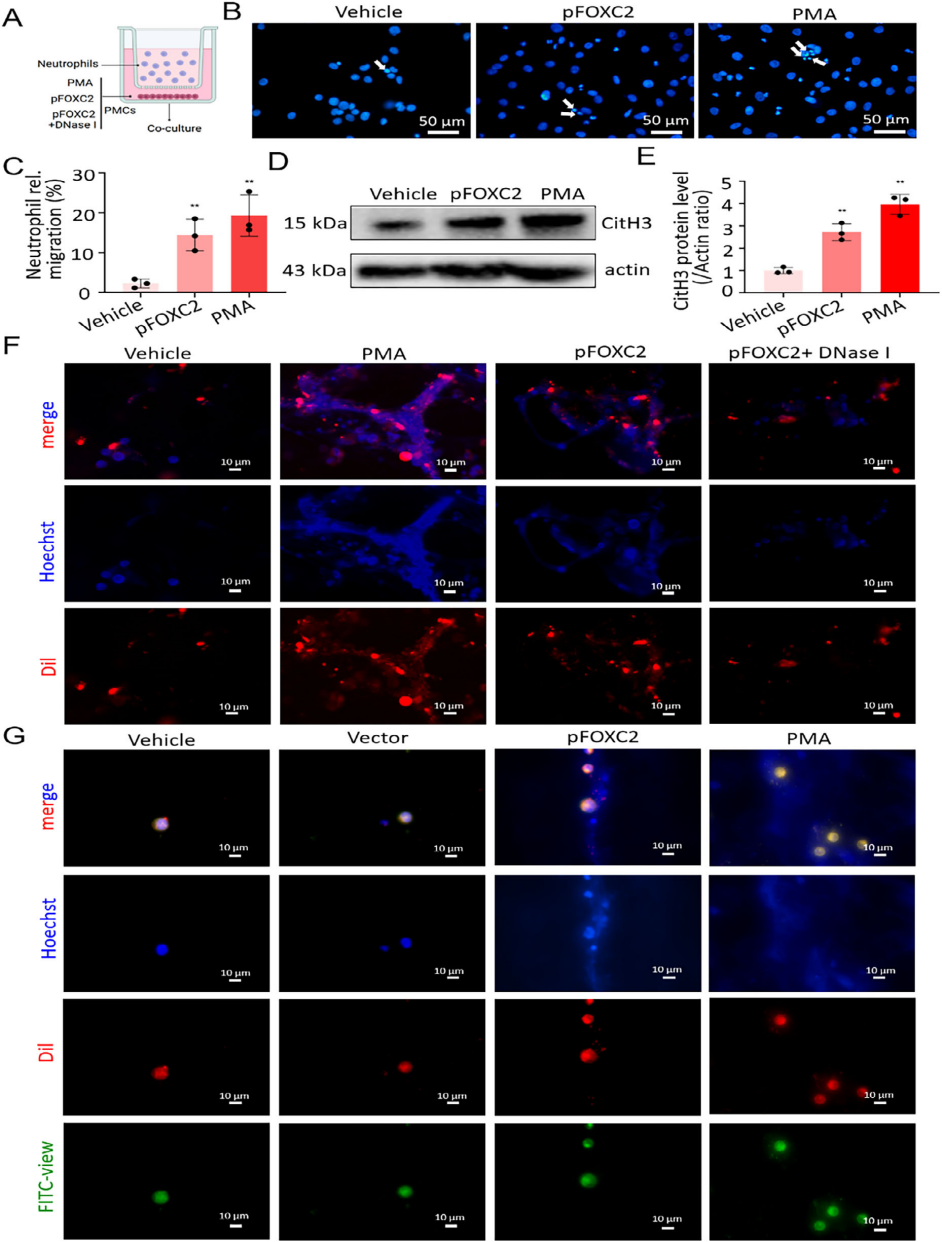

**Figure:**
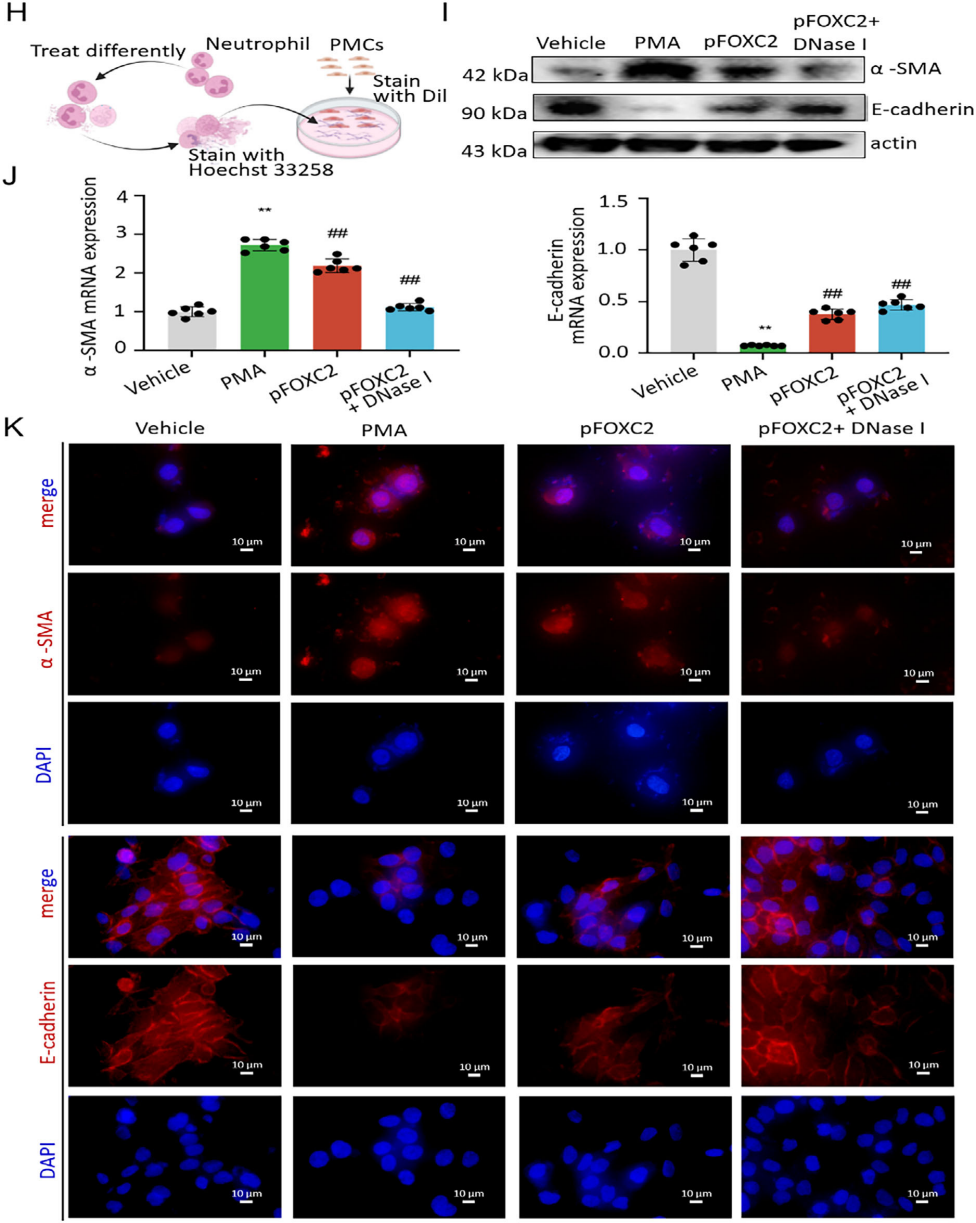

### 2.6 An Anti‐Fibrotic Compound, Aloe‐Emodin, Diminishes FOXC2‐Induced Fibrotic Responses and Reverses NETs‐Induced EMT Through the TGF‐β1‐Smad2/3 Pathway in PMCs

**Figure 6:**
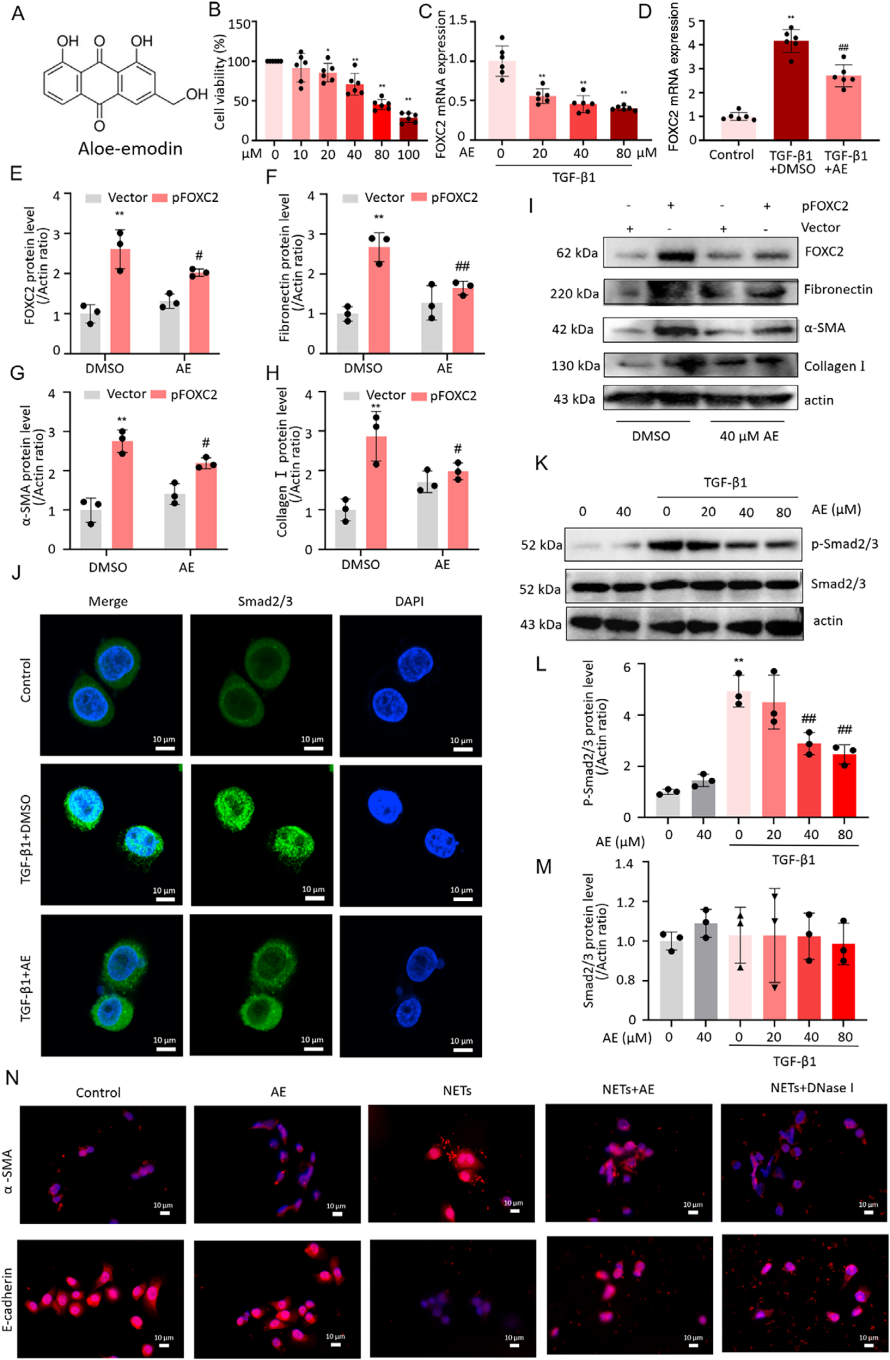
AE diminishes FOXC2‐induced fibrotic responses and reverses NETs‐induced EMT through the TGF‐β1‐Smad2/3 pathway in vitro. A) The molecular structure formula of AE. B) The cell viability of PMCs treated with different concentrations of AE (10, 20, 40, 80, and 100 µM) for 24 h by CCK8 assay. n = 6 per group. Compared with the control group, ^**^ *p* < 0.01, ^*^ *p* < 0.05. C) qRT‐PCR analysis of FOXC2 expression in TGF‐β1‐pretreated PMCs upon different concentrations of AE (20, 40, and 80 µM) for 24 h. n = 6 per group. Compared with the control group, ^**^ *p* < 0.01. D) qRT‐PCR analysis of FOXC2 expression in PMCs among different groups. n = 6 per group. Compared with the control group, ^**^ *p* < 0.01. Compared with the TGF‐β1+DMSO group, ^##^ *p* < 0.01. E–I) Western blot analyses of protein levels of FOXC2, Fibronectin, α‐SMA, and Collagen I in PMCs pre‐transfected with empty or pFOXC2 vector following treatment with AE for 24 h. n = 3 per group. Compared with the vector group, ^**^ *p* < 0.01. Compared with pFOXC2 group, ^#^ *p* < 0.05. J) Immunofluorescence staining images of Smad2/3 (green) in TGF‐β1‐pretreated PMCs upon 40 µM AE treatment for 24 h. Scale bar = 10 µm. K–M) Western blot analyses of protein levels of Smad2/3 and p‐Smad2/3 in TGF‐β1‐pretreated PMCs upon different concentrations of AE for 24 h. n = 3 per group. Compared with the control group, ^**^ *p* < 0.01. Compared with the TGF‐β1 group, ^##^ *p* < 0.01. N) Immunofluorescence staining images of mesenchymal‐related marker (α‐SMA; stained with red) and mesothelial‐related marker (E‐cadherin; stained with red) expressions among different groups. Scale bar = 10 µm.

It is said that FOXC2 may drive wound healing and fibrotic tissue formation.^[^ ^36^ ^]^ Given its critical role in TGF‐β1‐stimulated PMCs, we further investigate the feasibility of targeting FOXC2 with AE. AE is one of the main active ingredients of the herbal medicine *HouXueTongFu* formula in preventing PPA, as evidenced by our previous studies.^[^ ^20^, ^21^ ^]^ The molecular structure formula of AE was presented in **Figure** **6**A. The viability of PMCs decreased in a dose‐dependent manner with increasing concentrations of AE (Figure 6B). Consistently, qRT‐PCR analysis revealed that FOXC2 mRNA expression in TGF‐β1‐treated PMCs was also suppressed by AE in a dose‐dependent manner (Figure 6C). 40 µM AE treatment reduced the viability of PMCs to 70.9% of the control group (*p* < 0.01), and this concentration was therefore selected for subsequent experiments. We found that AE treatment could significantly reduce the mRNA expression of FOXC2 (2.7 ± 0.5) when compared with the TGF‐β1 group (1.0 ± 0.2, *p* < 0.01), as shown in Figure 6D. Western blot results indicated that FOXC2 overexpression remarkably promoted the synthesis of Fibronectin, α‐SMA, and Collagen I, which were significantly reversed upon AE treatment (Figure 6E–I). Immunofluorescent staining demonstrated that Smad2/3 was accumulated in the nuclei after treatment with TGF‐β1, whereas this accumulation was reversed by AE (Figure 6J). Moreover, TGF‐β1 induced the phosphorylation of Smad2/3. Nevertheless, this process was inhibited by AE (Figure 6K–M). To further investigate the potential impact of AE on NETs‐induced EMT, PMCs were cocultured with PMA‐induced NETs and subsequently treated with or without AE. We found that NETs could facilitate the EMT process in PMCs, as shown by an increase in the expression of mesenchymal‐related marker (α‐SMA) and a decrease in the expression of mesothelial‐related marker (E‐cadherin). However, AE treatment reversed these effects (Figure 6N). Collectively, the above results suggested that AE might be an anti‐fibrotic compound by diminishing FOXC2‐induced fibrotic responses and reversing NETs‐induced EMT through the TGF‐β1‐Smad2/3 pathway in PMCs.

### 2.7 Administration of Aloe‐Emodin Attenuates Peritoneal Adhesion by Inhibiting FOXC2‐Driven Fibrotic Responses and Regulating NETs Formation and EMT Changes Through the TGF‐β1‐Smad2/3 Pathway In Vivo

**Figure 7:**
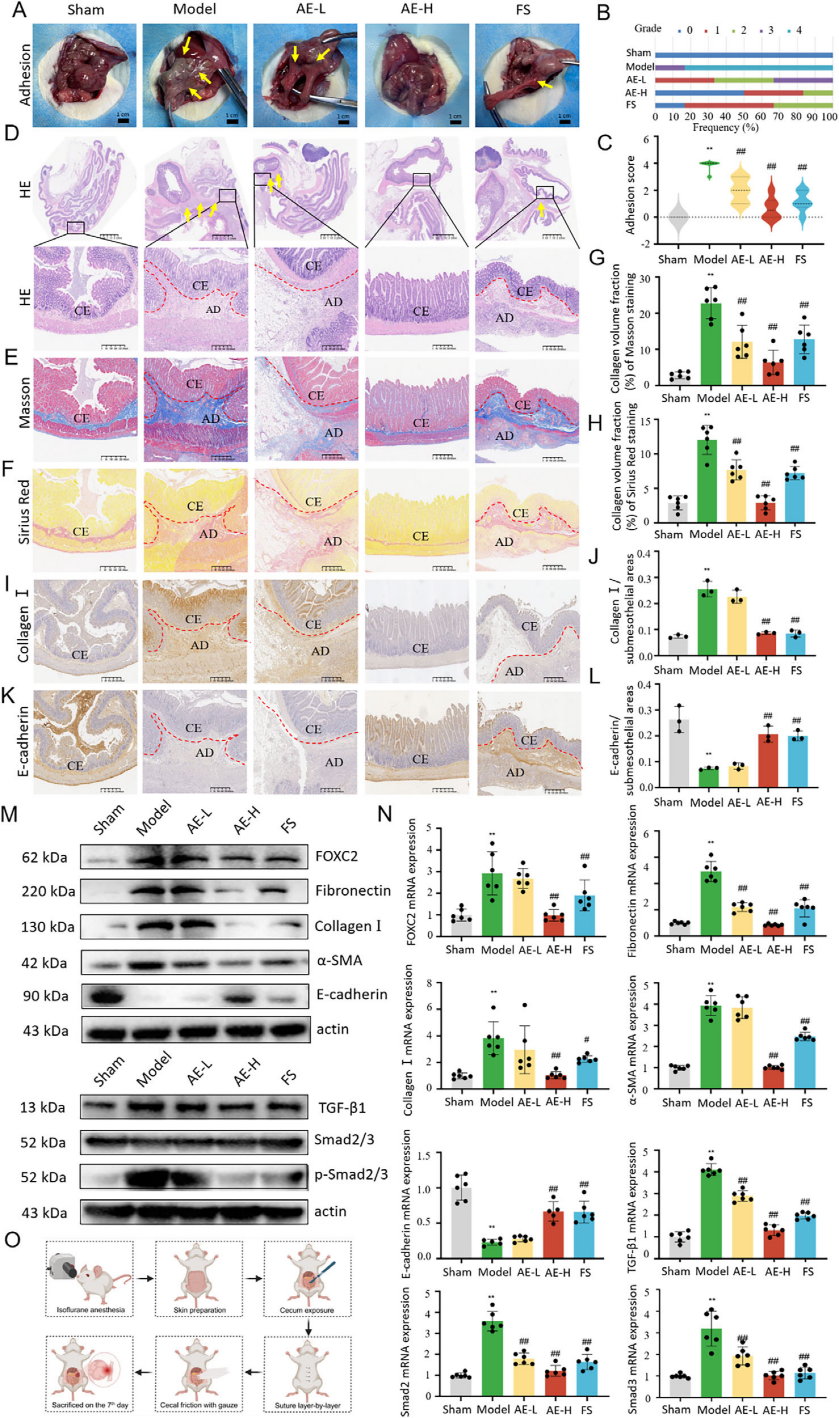
AE attenuates peritoneal adhesion by inhibiting FOXC2‐driven fibrotic responses and regulating the EMT changes through the TGF‐β1‐Smad2/3 pathway in vivo. A) Representative images of adhesion formation in different treated groups. Yellow arrows showed the adhesion between the abdominal wall and cecum in gross observation. Scale bar = 1 cm. B) Adhesion frequency of different treated groups. n = 6 per group. C) Adhesion scores of different treated groups. n = 6 per group. Compared with the sham group, ^**^ *p* < 0.01. Compared with the model group, ^##^ *p* < 0.01. D) H&E staining of the cecum section in different treated groups. CE: cecum, AD: adhesion. Scale bar = 400 µm. E) Masson staining of the cecum section in different treated groups. CE: cecum, AD: adhesion. Scale bar = 400 µm. F) Sirius Red staining of the cecum section in different treated groups. CE: cecum, AD: adhesion. Scale bar = 400 µm. G) Collagen volume fraction of Masson staining of the cecum section in different treated groups. n = 6 per group. Compared with the sham group, ^**^ *p* < 0.01. Compared with the model group, ^##^ *p* < 0.01. H) Collagen volume fraction of Sirius Red staining of the cecum section in different treated groups. n = 6 per group. Compared with the sham group, ^**^ *p* < 0.01. Compared with the model group, ^##^ *p* < 0.01. I) Immunohistochemical images of Collagen I of the cecum section in different treated groups. CE: cecum, AD: adhesion. Scale bar = 400 µm. J) Quantitative analysis of immunohistochemical staining intensity for Collagen I in the cecum section of different treated groups. n = 3 per group. Compared with the sham group, ^**^ *p* < 0.01. Compared with the model group, ^##^ *p* < 0.01. K) Immunohistochemical images of E‐cadherin of the cecum section in different treated groups. CE: cecum, AD: adhesion. Scale bar = 400 µm. L) Quantitative analysis of immunohistochemical staining intensity for E‐cadherin in the cecum section of different treated groups. n = 3 per group. Compared with the sham group, ^**^ *p* < 0.01. Compared with the model group, ^##^ *p* < 0.01. M) Western blot analyses of protein abundance of FOXC2, Collagen I, mesenchymal‐related proteins (Fibronectin, α‐SMA), mesothelial‐related protein (E‐cadherin), TGF‐β1, Smad2/3, and p‐Smad2/3 in different treated groups. N) qRT‐PCR analysis of mRNA expressions of FOXC2, Collagen I, mesenchymal‐related markers (Fibronectin, α‐SMA), mesothelial‐related marker (E‐cadherin), TGF‐β1, Smad2, and Smad3 in different treated groups. n = 6 per group. Compared with the sham group, ^**^ *p* < 0.01. Compared with the model group, ^##^ *p* < 0.01, ^#^ *p* < 0.05. O) Schematic diagram of the PPA model preparation.

To evaluate the critical effect of AE on peritoneal adhesion formation, a PAA model was prepared and treated with daily intragastric administration of AE for 7 days. We found that the wounds of all rats healed properly, without any signs of infection or complications during the process of model preparation. No rats died, and the body weights remained consistent across the five groups with no significant differences. All rats were euthanized on the 7th day following the successful establishment of the model. The representative images of peritoneal adhesion in five groups were illustrated in **Figure** **7**A. The frequency of different grades and adhesion scores among groups was shown in Figure 7B,C, respectively. Severe adhesion between the cecum and adjacent organs was observed in the PAA model, whereas less adhesion was found in the AE‐H group. The adhesion scores were listed as follows: model > AE‐L > FS > AE‐H > sham. Compared to the sham group, the model group exhibited the highest levels of adhesion scores. However, the incidence and severity of adhesions were significantly reduced in the groups treated with AE or FS. H&E, Masson, and Sirius red staining revealed that the PAA model exhibited significantly increased adhesion thickness and extensive collagen deposition in the abraded cecum when compared with the sham group. However, treatment with AE‐H resulted in a dramatic reduction in both adhesion thickness and collagen deposition compared to the model group (Figure 7D,F). Similar results could be found in the semi‐quantitative assay of Masson staining and Sirius red staining (Figure 7G,H). The results of the immunohistochemical analysis showed that the staining intensity of collagen I in the model group was stronger than that in the sham group. However, the staining intensity of collagen I was significantly reduced in the AE‐H group in comparison with the model group (*p* < 0.01), as presented in Figure 7I,J. In contrast, there was a significant reduction in the expression of E‐cadherin in the PPA model when compared to the sham group (*p* < 0.01). After treatment with AE‐H, the staining intensity of E‐cadherin was markedly higher than that of the model group (Figure 7K,L). In comparison with the sham group, the synthesis of mesenchymal‐related proteins (Fibronectin, α‐SMA), Collagen I, and FOXC2 was significantly upregulated in the model group. After treatment with AE‐H, the abundance of these proteins was markedly downregulated when compared with the model group. In contrast, the protein level of mesothelial‐related protein (E‐cadherin) was notably lower than that of the model group. After treatment with AE‐H, the E‐cadherin protein abundance was increased markedly in comparison with the model group (Figure 7M). The results were in line with those of qRT‐PCR (Figure 7N). Moreover, when compared with the sham group, the protein levels of TGF‐β1 and p‐Smad2/3 were elevated in the model group. After treatment with AE‐H, the protein levels of TGF‐β1 and p‐Smad2/3 were notably decreased (Figure 7M). The results were consistent with those of qRT‐PCR (Figure 7N). The schematic diagram of the PPA model preparation was displayed in Figure 7O.

**Figure 8:**
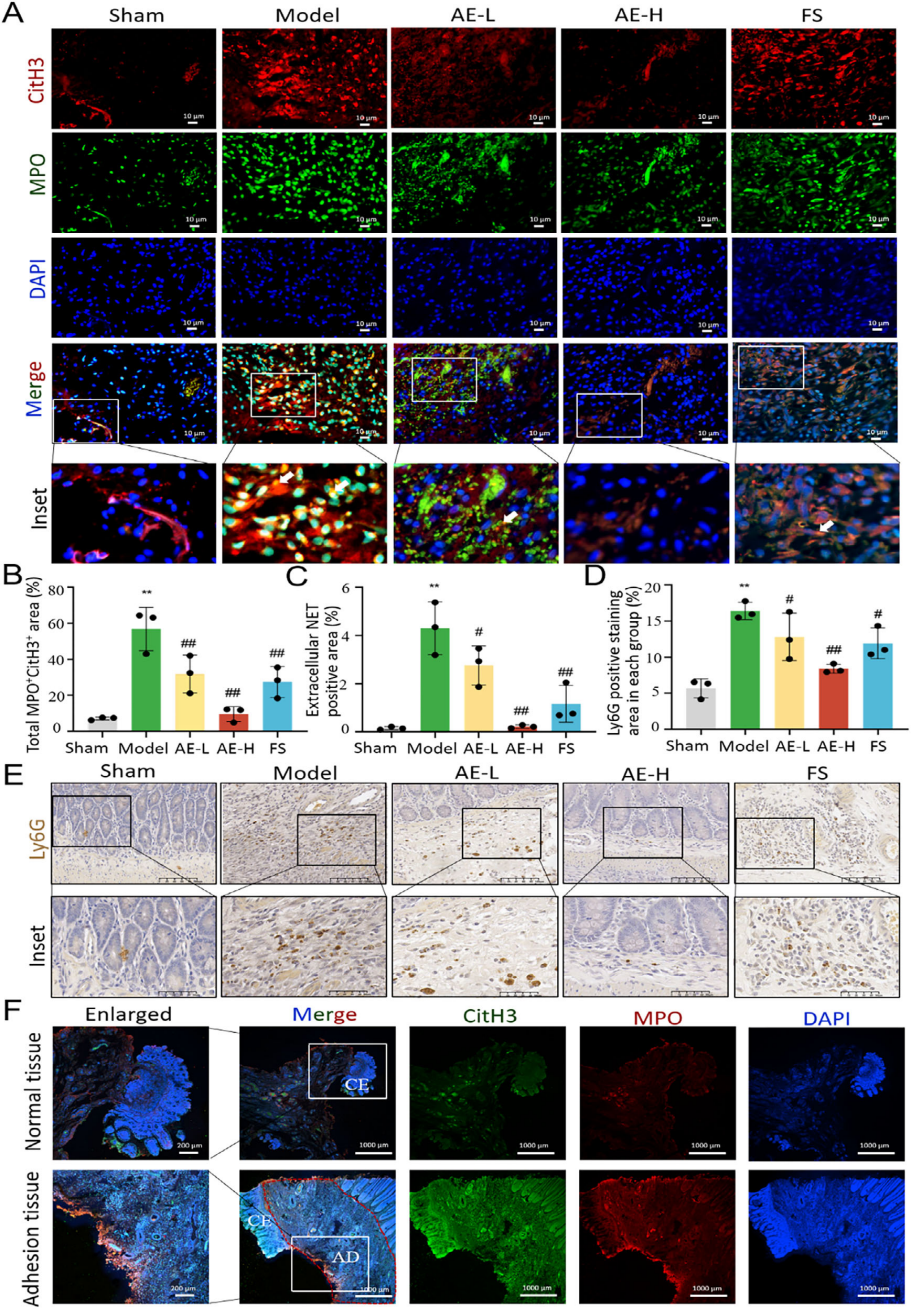
AE attenuates peritoneal adhesion by reducing NETs formation. A) Immunofluorescence staining images of NETs in the rats' cecum section of different treated groups, labeled by using MPO (green) and CitH3 (red) markers. Scale bar = 10 µm. B) Quantitative analysis of fluorescence intensity of MPO^+^ CitH3^+^ double‐positive staining area in rats' cecum sections of different treated groups. n = 3 per group. Compared with the sham group, ^**^ *p* < 0.01. Compared with the model group, ^##^ *p* < 0.01. C) Quantitative analysis of fluorescence intensity in the extracellular NETs staining area of rats' cecum sections in different treated groups. n = 3 per group. Compared with the sham group, ^**^ *p* < 0.01. Compared with the model group, ^##^ *p* < 0.01, ^#^ *p* < 0.05. D) Quantitative analysis of immunohistochemical intensity in the Ly6G‐positive staining area of rats' cecum sections in different treated groups. n = 3 per group. Compared with the sham group, ^**^ *p* < 0.01. Compared with the model group, ^##^ *p* < 0.01, ^#^ *p* < 0.05. E) Immunohistochemical images of Ly6G in rats' cecum sections in different treated groups. Scale bar = 100 µm. F) Immunofluorescence staining images of NETs in human ileostomy tissue, labeled by using CitH3 (green) and MPO (red) markers. Scale bar = 1000 µm in unenlarged images. Scale bar = 200 µm in enlarged images. CE: cecum, AD: adhesion.

In addition, the co‐localization of myeloperoxidase (MPO; a neutrophil marker) and CitH3 (a NETs marker) was employed to label NETs on the rats' adherent cecum. The total MPO^+^ CitH3^+^ area and extracellular NETs (web‐like structures) were calculated, respectively. The MPO^+^ CitH3^+^ double‐positive staining area, accounting for ≈56.8 ± 12.0% in the model group, was significantly higher than that in the sham group with a proportion of 7.1 ± 0.7% (*p* < 0.01). This proportion was reduced to 9.6 ± 4.1% in the AE‐H group (*p* < 0.01). Similarly, the extracellular NETs area in the model group reached 4.3 ± 1.0%, which was significantly higher than that in the sham group (0.2 ± 0.1%, *p* < 0.01). AE‐H treatment could reduce this percentage to 0.2 ± 0.1%, as presented in **Figure** **8**A–C. Ly6G, a surface marker of neutrophil activation, is associated with enhanced NETs generation capacity, particularly under inflammatory or specific stimuli.^[^ ^37^ ^]^ The immunohistochemical analysis further verified the above findings. As shown in Figure 8D,E, the Ly6G^+^ staining area with a proportion of 16.4 ± 1.2% in the model group was notably higher than that in the sham group with a proportion of 5.7 ± 1.3% (*p* < 0.01). This ratio was markedly reduced to 8.4 ± 0.6% in the AE‐H group. Immunofluorescence staining images of NETs in human ileostomy tissue exhibited similar morphological patterns to those observed in the adherent cecum of rats (Figure 8F). Taken together, the above discovery suggested that administration of AE could attenuate peritoneal adhesion by inhibiting FOXC2‐driven fibrotic responses and regulating NETs formation and EMT changes through the TGF‐β1‐Smad2/3 pathway in vivo.

### 2.8 Aloe‐Emodin Binds Directly to FOXC2, and Ser125 Residue is Critical for the Binding of FOXC2 to Aloe‐Emodin

**Figure 9:**
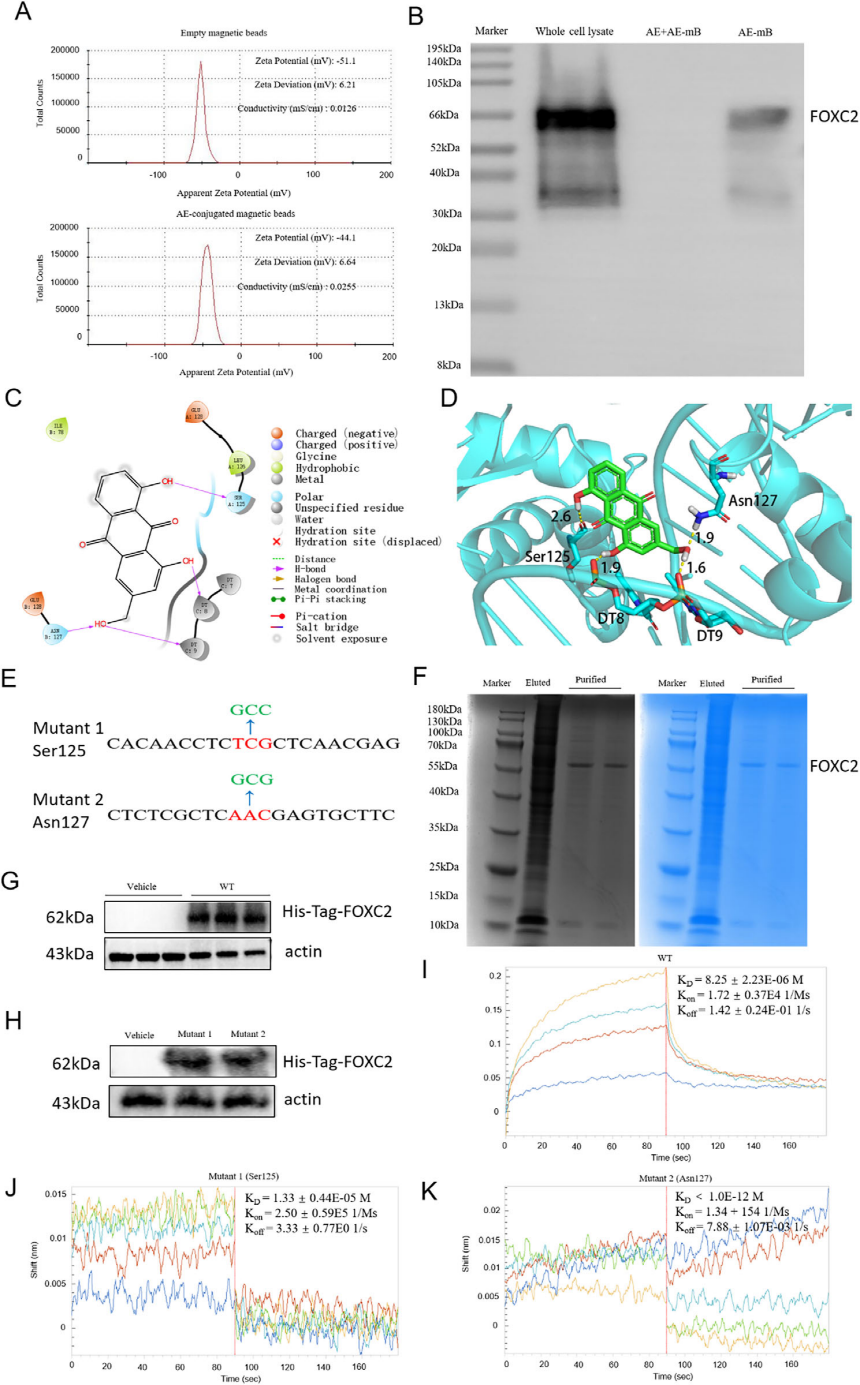
Aloe‐emodin binds directly to FOXC2, and the Ser125 residue is critical for the binding of FOXC2 to aloe‐emodin. A) The zeta potential of the empty magnetic beads and the AE‐conjugated magnetic beads. B) Western blot analyses of FOXC2 abundance in the whole cell lysate, AE+AE‐mB (AE‐conjugated magnetic microbeads), and AE‐mB groups. C,D) 2D and 3D views of the molecular docking assay of AE and FOXC2, respectively. Potential binding pocket residues were predicted. E) The diagram of the site‐directed mutagenesis technique targeting two amino acid residues (Ser125 and Asn127) in the binding pocket. F) Grayscale and Coomassie blue results of purified FOXC2 expression. G,H) Western blot analyses of His‐tagged FOXC2 expression levels of vehicle, wild‐type (WT), and different mutant groups. I) The binding of AE to FOXC2 (WT) was determined by BLI assay. J) The binding of AE and mutant 1 (Ser125) was determined by BLI assay. K) The binding of AE and mutant 2 (Asn127) was determined by BLI assay.

To confirm the binding of AE to FOXC2, we conducted a magnetic beads pull‐down assay in PMCs. The zeta potential of empty magnetic beads was −51.1 mV, and the coupled magnetic beads (AE‐conjugated magnetic beads) was −44.1 mV by a particle size analyzer (Anton Paar, Austria), as shown in **Figure** **9**A. The shift in charge between the empty and AE‐conjugated magnetic beads suggested that the carboxyl groups present on the magnetic beads' surface were occupied by AE, thus validating the successful fabrication of the AE‐conjugated magnetic beads. There is a strong band in the whole cell lysate group, which served as a positive control. The absence of the target band in the group pre‐incubated with 6 mmol free AE (dissolved in 5% DMSO) before AE‐conjugated magnetic bead treatment, whereas the presence of a weak band in the group incubated with AE‐conjugated magnetic beads alone, confirms that AE could competitively bind to FOXC2 in PMCs, as displayed in Figure 9B. To further predict the binding mode of AE and FOXC2, the Schrödinger molecular modeling suite (version 2021–2), Glide docking, and SP precision were employed and four hydrogen bond interactions were observed. The hydroxyl group at position 1 formed a polar hydrogen bond with the phosphate group of DT8 on the DNA chain, at a distance of 1.9 Å. The hydroxymethyl group at position 3 simultaneously formed two polar hydrogen bonds with the amino group of the Asn127 side chain and the phosphate group of DT9 on the DNA chain, at distances of 1.9 and 1.6 Å, respectively. The hydroxyl group at position 8 formed a polar hydrogen bond with the carbonyl group on Ser125, at a distance of 2.6 Å. 2D and 3D structures were shown in Figure 9C,D, respectively. Then, the site‐directed mutagenesis technique targeting key amino acid residues in the binding pocket was used to validate the molecular interactions. To understand the impact of amino acid codon mutations at the protein‐coding level, the serine at position 125 was mutated into alanine (Ser125, mutant 1), and the asparagine at position 127 was mutated into alanine (Asn127, mutant 2), as listed in Figure 9E. The FOXC2 recombinant expression vector was constructed, transfected into human embryonic kidney (HEK) 293T cells, and purified successfully, as displayed in Figure 9F. Western blot analyses further validated the high FOXC2 protein abundance in the wide‐type (WT) and different mutant groups (Figure 9G,H). Finally, the Biolayer interferometry (BLI) assay was conducted to calculate the rates of biomolecular kinetics to explore the specific binding effects. The affinity results between AE and WT FOXC2 indicated that AE had an affinity function for FOXC2 of (8.25 ± 2.23) × 10^−6^ M. The *K*_on_ and *K*_off_ values of AE for WT FOXC2 were (1.72 ± 0.37) × 10^−4^ 1/Ms, and (1.42 ± 0.24)× 10^−1^ 1/s, respectively (Figure 9I). The *K*_D_ value of mutant 1 (Ser125) was (1.33 ± 0.44) × 10^−5^ M, while the *K*_D_ value of mutant 2 (Asn127) was less than 1.0 × 10^−12^ M, as displayed in Figure 9J,K. The binding strength between AE and FOXC2 could be listed as follows: mutant 2 (Asn127) > mutant 1(Ser125). Altogether, the discoveries displayed that AE is directly bound to FOXC2, and mutant 1 exhibited a weaker interaction with AE compared to mutant 2, demonstrating that the Ser125 residue was critical for the binding of FOXC2 to AE.

## 3 Discussion

Mesothelium integrity has a significant impact on peritoneal adhesion. Mesothelial cells form a monolayer that covers the serosal surface of the peritoneum, serving as both a physical barrier and a functional regulator of the abdominal cavity. Upon peritoneal injury, pro‐fibrotic cytokines, including TGF‐β, are released. TGF‐β acts as a central mediator of fibrosis by activating the Smad2/3 signaling cascade, which drives peritoneal fibrosis through the induction of EMT in PMCs. Sustained TGF‐β signaling further promotes the transdifferentiation of PMCs into migratory fibroblast‐like cells. These phenotypically altered cells excessively produce ECM components such as collagen I and fibronectin, creating a fibrotic niche that disrupts tissue architecture. This pathological ECM remodeling exacerbates fibrosis and facilitates the development of peritoneal adhesions, characterized by abnormal fibrous bands between peritoneal surfaces.^[^ ^25^ ^]^ In this study, we observed that FOXC2 expression was upregulated upon TGF‐β1 exposure in a time‐dependent manner (Figure 1K,L), which indicated that FOXC2 might function as a key downstream effector of TGF‐β1‐mediated fibrotic responses in PMCs. Consistent with our findings, previous studies showed that FOXC2 was a downstream regulator of various cell types when pre‐treated with TGF‐β1.^[^ ^38^, ^39^, ^40^ ^]^ Of note, we found a positive feedback loop in which the TGF‐β1 expression could be augmented by FOXC2 overexpression (Figure 3J). The reciprocal regulation was in line with a previous study that FOXC2 interacts with the Smads to participate in the TGF‐β1‐Smad pathway^[^ ^19^ ^]^ and promotes the EMT process to drive cell responses after injury.^[^ ^18^ ^]^ These data highlight the critical pro‐fibrotic role of FOXC2 in TGF‐β1‐dependent fibrotic responses in PMCs. We also found that FOXC2 knockdown suppressed TGF‐β1‐induced Smad2/3 phosphorylation, whereas FOXC2 overexpression enhanced Smad2/3 phosphorylation levels in PMCs (Figure 4A–G), implying the critical roles of FOXC2 in the TGF‐β1‐Smad2/3 pathway. Moreover, a TGF‐β1‐Smad inhibitor further revealed that FOXC2 might function as a dual mediator and amplifier in TGF‐β1‐Smad2/3 signaling to drive EMT changes in PMCs (Figure 4J–M). Mechanistically, FOXC2 was involved in the process of ECM structural constituents in the peritoneum, evidenced by transcriptomic changes in FOXC2‐depleted PMCs (Figure 2). CRISPR/Cas9‐mediated FOXC2 knockdown attenuated EMT markers (upregulated E‐cadherin, p120, and downregulated Fibronectin; Figure 3E–H), whereas its overexpression exacerbated EMT‐driven phenotypic switching (Figure 3J,K). The results were consistent with previous studies, which suggested that silencing FOXC2 could significantly increase the p120 and E‐cadherin expression.^[^ ^41^, ^42^ ^]^ It is also said that FOXC2 was required for the maintenance of the mesenchymal phenotype after TGF‐β1‐induced EMT,^[^ ^43^ ^]^ and EMT will not be initiated in the absence of FOXC2.^[^ ^44^ ^]^ Given that the TGF‐β1‐Smad2/3 pathway serves as an important transcriptional activator of EMT in the fibrosis process,^[^ ^45^ ^]^ the above data collectively underscore the vital role of FOXC2 in TGF‐β1‐Smad2/3 pathway‐induced EMT and subsequent fibrotic progression in PPA.

Interestingly, we found that FOXC2 was also associated with the formation of NETs, evidenced by the KEGG enrichment analysis of RNA sequencing data in FOXC2‐depleted PMCs (Figure 2G). To address this issue, differently treated PMCs were cocultured with neutrophils. It showed that FOXC2 overexpression could attract neutrophil migration (Figure 5B,C). In parallel, the adhesive trapping assay illuminated that FOXC2 could promote the adhesion of PMCs by inducing neutrophils to form and release extensive web‐like NETs in vitro (Figure 5F,G). NETs have been shown to promote fibrosis by inducing EMT, thereby contributing to tissue fibrosis.^[^ ^46^ ^]^ To determine whether the following EMT progression was modulated, we analyzed EMT‐associated markers (α‐SMA and E‐cadherin) in PMCs after 24 h co‐culture with NETs. Consistent with our hypothesis, FOXC2 could mediate NETs‐induced EMT, as evidenced by increased α‐SMA expression and suppressed E‐cadherin levels. These findings further indicated that FOXC2 could facilitate NETs formation, thereby driving subsequent EMT changes. Our previous studies elucidated the involvement of the TGF‐β1‐Smad2/3 pathway,^[^ ^47^ ^]^ EMT changes,^[^ ^48^ ^]^ and NETs^[^ ^49^ ^]^ in the pathogenesis of peritoneal adhesion, as demonstrated in vivo or in vitro. However, these mechanisms have been investigated separately, and their interactions in driving adhesion formation have received limited attention. This research not only represented a pioneering endeavor to unravel the collective and synergistic role of these entities in the fibrosis progression of peritoneal adhesion but also pioneered the identification of FOXC2 as a linchpin through its synergistic effects between the TGF‐β1‐Smad2/3 signaling axis and NETs formation to subsequent EMT alteration during PPA pathogenesis. However, the dynamic spatiotemporal regulation of the reciprocal feedback between NETs and TGF‐β1/Smad2/3 signaling warrants further investigation.

More importantly, we found that AE, a natural anthraquinone derivative and the main active ingredient of the herbal medicine *Rheum palmatum L*.,^[^ ^22^ ^]^ which is one of the components of HXTF, exhibited anti‐fibrosis and anti‐adhesive effects on PPA. Based on molecular docking analysis revealing that AE successfully occupies the binding pocket of FOXC2, we hypothesized a direct physical interaction between AE and FOXC2. This interaction was further validated by competitive binding assays using magnetic bead pull‐down experiments. Subsequent site‐directed mutagenesis demonstrated that the Ser125 residue is essential for AE‐mediated activation of FOXC2. This finding not only provided insights into the molecular mechanism of AE‐FOXC2 interaction but also highlighted Ser125 as a potential site for designing more specific and effective inhibitors to modulate FOXC2 activity in a therapeutic context. In addition, the therapeutic effects of AE on PPA rodent models were examined. We found that AE could attenuate PPA formation, as evidenced by a significantly reduced adhesion score. AE could suppress collagen deposition, as indicated by the presence of fewer collagen fibers in the abraded cecum. This suppression might be achieved by inhibiting the TGF‐β1‐Smad2/3 signaling axis, blocking the formation of synergetic NETs, and thereby attenuating the alteration of EMT. All evidence suggested that AE might be regarded as a promising anti‐adhesion agent targeting FOXC2. However, our study also had several limitations. First, in the current study, we explored the critical role of the TGF‐β1‐Smads pathway in peritoneal fibrosis. While TGF‐β1 signaling is widely recognized as a canonical driver of fibrotic processes through both Smad‐dependent and non‐Smad pathways, our investigation was limited to the Smad axis. Future studies should explore the potential contribution of non‐Smad pathways to PPA formation, which may further refine therapeutic strategies targeting TGF‐β1 in fibrosis. Second, MC‐1‐F2 is the only identified experimental inhibitor of FOXC2, which can interact with the full length of FOXC2. However, only the DNA‐binding domain of FOXC2 has been resolved in a crystal structure.^[^ ^50^ ^]^ In our study, we found that AE could directly bind to FOXC2, and the Ser125 residue is essential for AE‐mediated activation of FOXC2. Further studies should explore how the interaction between AE and the full‐length FOXC2 influences the overall function of FOXC2 in fibrotic processes, and whether targeting the Ser125 residue could serve as a novel therapeutic strategy for PPA. Third, besides ECM receptor interaction, GSEA based on the GO database also revealed significant enrichment of expressed genes that were largely linked to the NF‐κB signaling pathway. In our prior research, we demonstrated that the TLR4/MyD88/NF‐κB pathway contributed to the pathogenesis of PPA.^[^ ^51^ ^]^ Nevertheless, in the current study, we did not thoroughly investigate the functional significance of the NF‐κB signaling pathway with FOXC2, nor did we explore the potential therapeutic effect of AE mediated through the NF‐κB signaling pathway. Fourth, biological gender differences may influence the pathophysiology of peritoneal adhesion. Future studies are warranted to incorporate both male and female rodent models to thoroughly investigate gender‐specific mechanisms and potential therapeutic effects.

**Figure 10:**
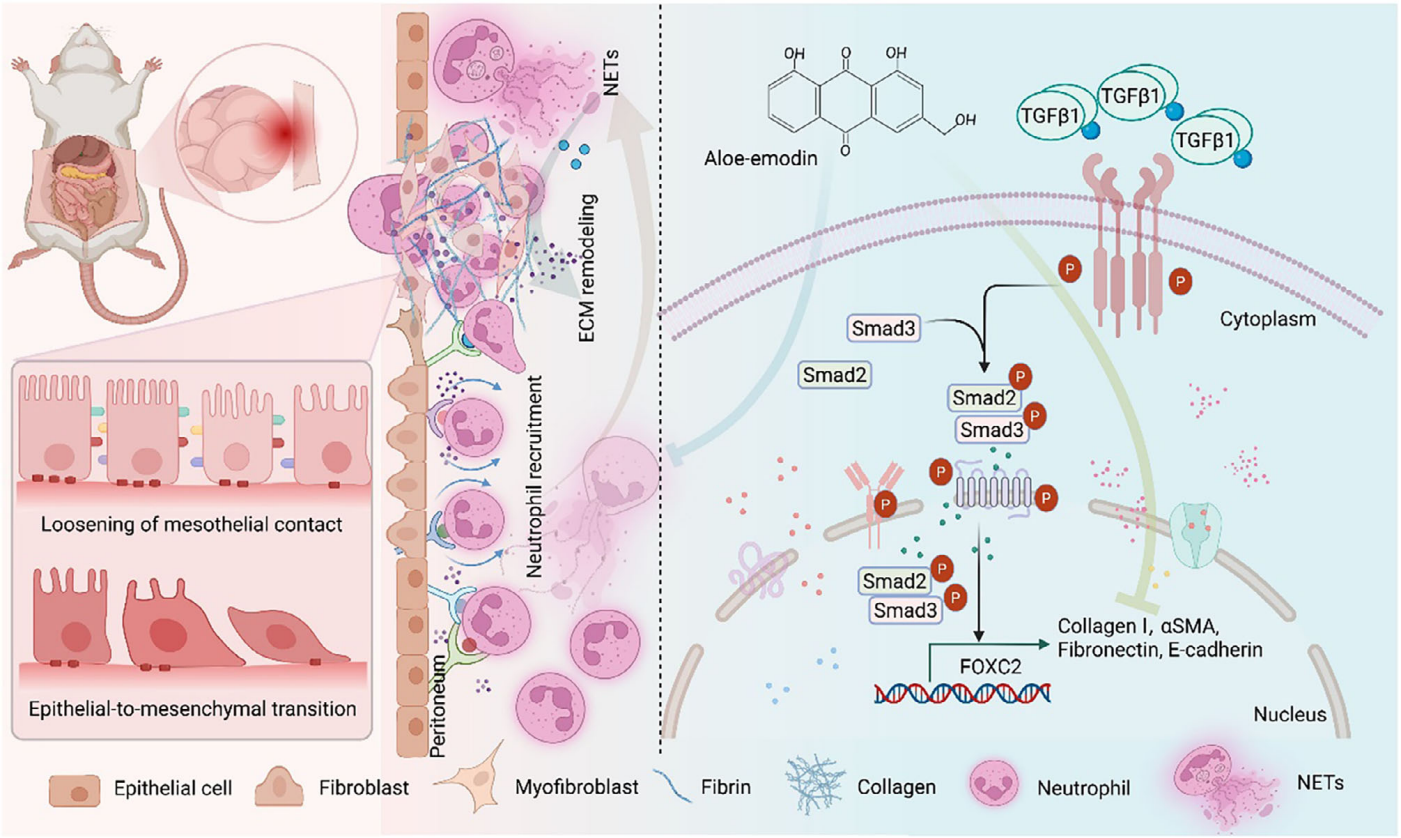
Schematic illustration of aloe‐emodin targeting FOXC2 disrupts NETs formation and subsequent EMT‐mediated postoperative peritoneal adhesion through TGF‐β1‐Smad2/3 pathway. Figures were created using Biorender.com (https://biorender.com/).

In summary, in the current study, we highlighted the critical role of FOXC2 as a pivotal effector in peritoneal adhesion formation. The aberrant overexpression of FOXC2 in human ileostomy tissue, rodent PPA rats, and TGF‐β1‐exposed PMCs correlated with NETs formation, subsequent EMT changes and the TGF‐β1‐Smad2/3 pathway. More importantly, AE could directly bind to the Ser125 residue of FOXC2. AE could also suppress FOXC2‐driven fibrotic responses and NETs formation to the following EMT process through the TGF‐β1‐Smad2/3 pathway (**Figure** **10**). All discoveries revealed that AE could serve as a promising compound for a FOXC2‐targeted therapeutic approach to attenuate postoperative peritoneal adhesion.

## 4 Experimental Section

### Materials

AE (S31411, purity ≥ 95%) was purchased from Yuanye Co., Ltd. The primary antibodies, such as α‐SMA (bs‐10196R, Bioss), Fibronectin (sc‐8422, Santa Cruz), FOXC2 (sc‐515472, Santa Cruz; 23066‐1‐AP, Proteintech), E‐cadherin (bs‐1519R, Bioss), p120 (sc‐23873, Santa Cruz), Claudin‐1 (sc‐137121, Santa Cruz), TGF‐β1 (sc‐130348, Santa Cruz), Smad2/3 (AF6367, Affinity Biosciences), p‐Smad2/3 (AF3367, Affinity Biosciences), collagen I (AF7001, Affinity Biosciences), CitH3 (NB100‐57135, Novus Biologicals), MPO (EM1901‐19, Huabio), Ly6G (MA1‐10401, Thermo Fisher Scientific), Histone H3 (17168‐1‐AP, Proteintech), and β‐actin (66009‐1‐Ig, Proteintech) were used. Second antibodies, such as anti‐rabbit IgG (Cy3, SA00009‐2, Proteintech), anti‐rabbit IgG (FITC, SA00003‐2, Proteintech), anti‐mouse IgG (Cy3, SA00009‐1, Proteintech), anti‐mouse IgG (FITC, SA00003‐1, Proteintech), HRP‐labeled Goat Anti‐Mouse IgG (A0216, Beyotime), and HRP‐labeled Goat Anti‐Rabbit IgG (A0208, Beyotime) were provided. Diamidino‐phenyl‐indole (DAPI; C1002, Beyotime), recombinant human TGF‐β1 (ck0023, Bioss), MC‐1‐F2 (HY‐149894, MCE), DGM (HY‐W005379, MCE), DMSO (D2650, Sigma–Aldrich), DNase I (D8071‐25, Solarbio), and PMA (P1585, Sigma–Aldrich) were used. TRIzol reagent (15596018CN) was purchased from Invitrogen (CA), and the qRT‐PCR kits (11141ES60, 11203ES08) were provided by Yeasen Co., Ltd. H&E staining kit (G1005) and Masson's trichrome staining kit (G1006) were bought from the Servicebio Co., Ltd., and a Sirius red staining kit (DC0041) was provided by Leagene Biotechnology Co., Ltd. Fluvastatin (FS) was bought from Novartis Pharmaceutical Co., Ltd.

### Cell Line and Culture

PMCs (HMrSV5) were bought from LMAI Bio Co., Ltd. The cells were cultured with RPMI 1640 medium (10‐040‐CV, Corning) containing 10% fetal bovine serum (11011‐8611, Every Green) and 100 U mL^−1^ penicillin/streptomycin/amphotericin in the incubator (ESCO, Singapore) under 5% CO_2_ at 37 °C. For TGF‐β1 treatment, PMCs were cultivated until they reached ≈70% confluence, deprived of serum for 4 h, and treated with 10 ng mL^−1^ recombinant human TGF‐β1 (ck0023, Bioss) for 24 h. For the in vivo experiment, PMCs were cultured in different concentrations of AE (20, 40, and 80 µm) pre‐dissolved in DMSO or cultured with FOXC2 inhibitor MC‐1‐F2 (20 µm) or TGF‐β1‐Smad inhibitor DGM (10, 20, 40 µm) for 24 h.

### Transfection of Plasmid

PMCs were transfected with AAV‐CMV‐HIS‐KMO plasmid containing full‐length FOXC2 (pFOXC2) synthesized by Tsingke Biotechnology Co., Ltd (Beijing) by using SuperKine Lipo3.0 (BMU111, Abbkine) according to the manufacturer's protocol.

### CRISPR/Cas‐Based FOXC2 Knockdown in PMCs

PMCs were transfected with a CRISPR/Cas‐based FOXC2 knockdown kit (KT504540, Cas9×3.0), which was bought from Haixing Biotechnology Co., Ltd, by using Lipofectamine 3000 (2369247, Thermo Fisher Scientific) according to the manufacturer's protocol. The stable knocked‐down PMCs were purified by using 20 µg mL^−1^ puromycin (ST551, Beyotime) and screened by high‐speed sorting‐type flow cytometry (FACSAriaTM Fusion, BD). Western blot and qRT‐PCR were employed to detect the knockdown effect of FOXC2 at both the protein and mRNA levels. Agarose gel electrophoresis and Sanger sequencing were utilized to verify the successful insertion of the sgRNA at the DNA level. The genomic editing PMCs were applied in the subsequent experiment.

### Cell Viability Assay

PMCs were seeded into 96‐well plates and allowed to attach for 12 h. Then, cells were exposed to a defined concentration range of DGM (5, 10, 20, 40, 80, and 160 µm) or AE (10, 20, 40, 80, and 100 µM) for 24 h. Finally, 10 µL of CCK8 solution was added to each well, and cell viability was assessed using a microplate reader (Tecan, Switzerland).

### Isolation of Neutrophils and Culture

Human peripheral blood was harvested into an EDTA anticoagulant tube, which was gently layered onto a 15 mL centrifuge tube containing 5 mL of Polymorphprep (AXS‐1114683, Serumwerk), followed by centrifugation at 500 × g for 30 min. The layer containing neutrophils was collected into a new centrifuge tube and washed with phosphate‐buffered saline (PBS). After centrifugation (450 × g, 5 min), red blood cells were lysed using a red blood cell lysis buffer (C3702, Beyotime). Subsequently, the isolated neutrophils were suspended in the RPMI 1640 medium (10‐040‐CV, Corning) for the subsequent experiment. The experimental procedures have been approved by the Institutional Ethics Committee of Ningbo Municipal Hospital of TCM (No. KYSL‐2024‐001‐074).

### Transwell Migration Assay

Migration assays were conducted using 24‐well plates with 8‐µm Transwell chambers (3422, Corning). PMCs were digested, resuspended, and adjusted to a cell density of 3 × 10^5^ cells mL^−1^ in the lower chamber. Then, cells were treated with 50 ng mL^−1^ PMA (P1585, Sigma‐Aldrich; 4 h), pFOXC2 (24 h) with or without DNase I (D8071‐25, Solarbio; 4 h), respectively. Finally, 200 µL of cell suspension containing neutrophils was added to the upper chamber to co‐culture for another 2 or 4 h in a 37 °C, 5% CO_2_ incubator (ESCO, Singapore). Cells containing PMCs with migrated neutrophils in the lower chamber were used for the following experiment.

### Cell Adhesion Assay

1 × 10^6^ neutrophils were plated in a 24‐well plate and stimulated with PMA, pFOXC2, empty vector, or pFOXC2+DNase I for 4 h, respectively. 5 × 10^5^ PMCs or FOXC2‐KD PMCs were stained with Dil (C1036, Beyotime) for 15 min. Then, Dil‐labeled cells were added to the wells and cultured for 30 min at 37 °C in a 5% CO_2_ atmosphere. After three PBS washes and 4% paraformaldehyde fixation, the nucleus was stained with Hoechst 33258 (C1017, Beyotime) for 10 min. The attached cells were visualized under a fluorescence microscope (Olympus, Japan).

### NETs Formation and EMT Induction in Monoculture

Neutrophils were seeded at a density of 5 × 10^5^ cells per well onto glass coverslips placed in 12‐well plates. The cells were activated with 50 ng mL^−1^ PMA (P1585, Sigma–Aldrich) for 4 h. DNase I (0.25 mg mL^−1^) was used as a control to inhibit NETosis. NETs were collected and used to induce EMT, as described in a previous study.^[^ ^14^ ^]^ Briefly, PMCs were cultured onto glass coverslips placed in 12‐well plates. NETs were added to each well, and PMCs were then treated with or without 40 µM AE for 24 h. The cells were fixed with pre‐cooled 4% paraformaldehyde for subsequent immunofluorescent staining.

### Sirius Red Staining (Direct Red 80)

The presence and quantification of collagen deposition were determined histologically by Sirius Red staining. Specifically, PMCs were treated with vector or pFOXC2 plasmid for 24 h, respectively. Subsequently, the cells were stained with a Sirius Red staining kit according to the manufacturer's guidelines. For rectum sections, the slides were immersed in a 0.1% Sirius Red solution for 1 h and then rinsed with 0.5% hydrochloric acid. The collagen appeared red, whereas the non‐collagenous components appeared orange.

### RNA Sequencing (RNA‐Seq)

After extracting total RNA from PMCs using the TRIzol method, RNA integrity was assessed with the Agilent 2100 Bioanalyzer, ensuring RIN values exceeded 8.0. Before library construction, ribosomal RNA was depleted using the NEBNext rRNA Depletion Kit. Strand‐specific RNA‐seq libraries were then generated using the NEBNext Ultra II Directional RNA Library Prep Kit, involving steps of cDNA synthesis, end repair, adapter ligation, and PCR amplification. Sequencing was performed on the Illumina NovaSeq 6000 platform, generating 150 bp paired‐end reads with over 40 million reads per sample. Raw data were processed to obtain clean data by removing irrelevant information, such as adapters, using internal Perl scripts. The overall quality of the RNA‐seq data was evaluated using Qualimap. The EdgeR R package (version 3.12.1) was used to analyze and screen differentially expressed genes, while the clusterProfiler R package was employed for GO annotation, KEGG enrichment, and GSEA analysis.

### Preparation of Aloe‐Emodin‐Conjugated Magnetic Microbeads

The AE‐conjugated magnetic microbeads were prepared as follows: carboxyl‐functionalized magnetic beads (10 mg, Yike Biological Co., Ltd., China) were dispersed in 1 mL anhydrous CH_2_Cl_2_ under argon. To this suspension, 30 mg 4‐(Dimethylamino) pyridine Novabiochem (Sigma‐Aldrich) and 5 mg AE were added, followed by dropwise addition of N, N'‐dicyclohexylcarbodiimide (Sigma‐Aldrich) at 0 °C. After 5 min activation at 0 °C, the mixture was stirred at 20 °C for 3 h under anhydrous conditions. The crude product was washed with 0.5M  HCl and saturated NaHCO_3_ solution, dried with MgSO_4_, and concentrated to obtain crystalline AE‐coupled conjugates. The conjugates were then blocked with 0.3% casein in phosphate‐buffered saline containing 0.05% Tween‐20 (PBST, pH 7.4) for 1 h to minimize nonspecific binding and stored in 0.25% BSA‐PBS (pH 7.4) at 4 °C for long‐term stability. The surface charge of the empty and AE‐conjugated magnetic microbeads was determined by a particle size analyzer in a zeta potential mode and applied to identify the binding interaction of AE to FOXC2 through the pull‐down assays.

### Molecular Docking

The Discovery Studio 3.0 (DS 3.0) molecular modeling suite, including the CDOCKER docking module and SP precision, was employed to investigate the interaction between FOXC2 and AE. The structure of AE was created using ChemBioDraw Ultra 14.0, saved in mol format, and subsequently processed in DS 3.0. It was then subjected to energy minimization, employing 2000 steps of the steepest descent method followed by 2000 steps of the conjugate gradient method. The 3D structure of the receptor protein was obtained from the Protein Data Bank (PDB ID: 6O3T) and prepared using the “prepare protein” module in DS 3.0 with default settings. Molecular docking simulation was conducted using the CDOCKER module in DS 3.0. The binding site was defined based on a radius of 10 Å, with all other parameters set to default. Intermolecular interactions were analyzed using the PyMOL Molecular Graphics System (Version 2.2.2).

### Protein Overexpression and Purification

The plasmids containing FOXC2 (WT), and FOXC2 mutants (Ser125, the serine at position 125 was mutated into alanine; Asn127, the asparagine at position 127 was mutated into alanine) synthesized by Tsingke Biotechnology Co., Ltd (Beijing) were transfected into HEK293T cells by using SuperKine Lipo3.0 (BMU111, Abbkine) according to the manufacturer's protocol. The cell culture medium was discarded, and RIPA lysis buffer (P0013B; Beyotime) was added to the culture dish to lyse the cells. The cell lysate was then collected and ultrasonicated on ice for 2 min to prevent overheating, followed by incubation in an ice–water bath for another 30 min to ensure complete lysis. After the lysate was centrifuged at 4 °C, 15000 rpm for 5 min, the supernatant containing recombinant His‐tagged FOXC2 was carefully harvested and applied onto HisTrap HP Ni‐NTA columns (GE17‐5247‐01, Cytiva). The protein was purified according to the instructions.

### Biolayer Interferometry (BLI) Assay

BLI is an entirely optical technique employed for characterizing the binding affinity between small molecules and wild‐type or mutant forms, which was conducted on an Octet RED 96 system (ForteBio).^[^ ^52^ ^]^ Briefly, 200 µL sample volumes were added to each well of opaque 96‐well plates. His‐tagged FOXC2 was loaded onto Ni‐NTA biosensors, which had been pre‐equilibrated in kinetics buffer (1× PBS containing 0.02% Tween‐20) at 30 °C. The biosensors were allowed to reach baseline in the kinetics buffer for 60 s. Subsequently, a concentration of AE solution (100, 50, 25, 12.5, and 6.25 µM in PBS buffer with 0.02% Tween‐20 and 0.5% DMSO) was immobilized onto the Ni‐NTA biosensors (ForteBio) for kinetics analysis. An equivalent amount of DMSO was added to the wells as controls. Following the association (60 s) and dissociation steps (90 s), the association and dissociation curves were fitted, and data were analyzed using Octet Data Analysis software. The affinity constant (*K*_D_) was determined as the ratio between the dissociation rate constant (*K*_off_) and the association rate constant (*K*_on_), that is, *K*_D_ = *K*_off_ / K_on_.

### Coomassie Blue Staining

The cell lysate was prepared using RIPA Lysis buffer (P0013B, Beyotime). The lysate was then loaded onto a 10% precast protein plus gel (36252ES10, Yeasen). After performing protein gel electrophoresis, the gel was transferred to a container filled with Coomassie blue staining solution (P0003S, Beyotime) and gently agitated for 1 h at room temperature. After washing with PBS three times, the protein bands on the gel were visualized using the Chemiluminescence Imaging System (Bio‐Rad, USA).

### Animal Procedures and Treatments

A total of thirty male Sprague–Dawley rats with a weight of (250 ± 10) g were supplied by Slaccas Experimental Animal Co., Ltd. (Shanghai). The animal experimental procedures were conducted following the Institutional and Local Committee on the Care and Use of Animals of Nanjing University of Chinese Medicine (approval No. 202401A035). The rats were randomly divided into five groups (n = 6 per group) that is the sham group, the model group, the AE‐L group, the AE‐H group, and the FS group (positive control^[^ ^53^ ^]^). Before the experiment, they were acclimatized for three days under standardized conditions, including a room temperature of 20 ± 2 °C, a relative humidity of 40 ± 5%, and a 12 h light/dark cycle.

The PPA model was prepared as described in the previous studies.^[^ ^20^, ^47^, ^48^ ^]^ In brief, rats were subjected to food deprivation for ≈12 h before the operation. During the operation, the rats were anesthetized with 1.5% isoflurane in a supine position. After the preoperative skin preparation, a 1.5 – 2 cm incision was made along the linea alba on the abdomen. The cecum was then gently pulled out tightly and scrubbed with sterile dry gauze until serosal petechiae appeared on the serosal layer. This process lasted for about 5 minutes. Subsequently, the abraded cecum was returned to its natural position within the abdomen, and the abdominal wall was sutured layer by layer. Rats in the sham group did not undergo any abrasive surgical procedure, except for a 5‐min exposure of the cecum to air. Rats in the other groups were prepared according to the model protocol.

In the AE‐L and AE‐H groups, 25 and 100 mg kg^−1^ of AE (dissolved in 0.5% CMC‐Na, C8620, Solarbio) were intragastrically administered, respectively. Both rats in the sham and model groups were intragastrically administered the same volume of 0.5% CMC‐Na. In the FS group, 10 mg kg^−1^ of FS was intragastrically administered. All treatments were given once a day for 7 days after the operation.

### Gross Observation and Adhesion Scoring

The adhesions were evaluated and scored by an observer who was unaware of the study design according to the five‐stage adhesion score, spanning from 0 to 4.^[^ ^54^, ^55^ ^]^ Adhesion severity was quantitatively evaluated using a standardized dual‐blind evaluation protocol based on established grading criteria: Grade 0 indicated complete absence of adhesions; Grade 1 encompassed 0–25% coverage by translucent, avascular filmy adhesions; Grade 2 extended this similar filmy adhesions involving 26–50% of the region; Grade 3 represented dense, opaque adhesions (51–75% coverage) containing capillary networks necessitating surgical division; Grade 4 denoted extensive vascularized fibrous bands (>75% coverage) with developed vasculature requiring precision dissection.

### Histopathological Staining

A cohort study involving 133 patients who underwent ileostomy suggested that ileostomy, a common surgical procedure, could be used as a model for peritoneal adhesion.^[^ ^56^ ^]^ Human ileostomy tissues (adhesion tissues and adjacent trimmed normal tissues) were collected from patients who underwent ileostomy surgery, and informed consent was obtained from all individuals. The study protocol was approved by the Institutional Ethics Committee of the Affiliated Hospital of Nanjing University of Chinese Medicine (approval No. 2024NL‐114‐01). Cecal tissue specimens from humans or rats were fixed in 4% neutral‐buffered formalin for 24 h, followed by paraffin embedding and sectioning into 4 µm‐thick histological slices. These sections were stained with H&E and Masson's trichrome for the assessment of morphological changes and collagen deposition. Stained samples were digitized using a whole‐side imaging system (KF‐Scan‐PL, Ningbo, China), followed by standardized blinded analysis, and randomized fields of view per slide were quantitatively evaluated under predefined acquisition parameters.

### Immunofluorescent Staining

Following sequential dewaxing and dehydration of cecal tissue sections, specimens were treated with QuickBlock blocking reagent (P0260, Beyotime) before overnight incubation at 4 °C with primary antibodies. After two 5‐min washes with PBS, sections were incubated with fluorochrome‐conjugated secondary antibodies for 60 min in the dark. Nuclear staining was subsequently performed using DAPI with a 5‐min incubation at room temperature.

As for PMCs, different treated cells were fixed with pre‐cooled 4% paraformaldehyde for 15 min and then stained with the primary antibodies for 2 h at room temperature. Afterward, cells were incubated with secondary antibodies for 1 h and stained with DAPI for another 5 min in the dark. The stained images were blindly imaged at random fields under a fluorescence microscope (Olympus, Japan), a laser scanning confocal microscope (Nikon AX, Japan).

### Histological Immunohistochemistry Staining

Cecal tissue sections were subjected to standard deparaffinization and graded ethanol rehydration before immunohistochemical processing according to the manufacturer's protocol for SP Rabbit & Mouse HRP Kit (DAB, CW2069, CWBIO). Briefly, the sections were incubated sequentially with primary antibodies in a humidity‐controlled chamber at 4 °C for 16 h. After washing with PBS, HRP‐conjugated species‐matched secondary antibodies were applied at room temperature (25 °C) for 10 min. Then, DAB substrate development was carried out for 5 min, followed by hematoxylin counterstaining for 30 s. Finally, the sections were subjected to dehydration through ethanol‐xylene gradients (70%, 95%, 100% ethanol; xylene). Images were performed using a whole‐slide imaging system (KF‐Scan‐PL, Ningbo, China), and randomized fields of view per slide were captured.

### Western Blot Analysis

Protein extraction was performed using RIPA Lysis Buffer (P0013B, Beyotime). The extracted proteins were quantified and then boiled in a water bath at 100 °C for 10 min with 1× SDS‐PAGE sample loading buffer (P0015A, Beyotime). Equal amounts of samples were loaded onto a 4–20% precast protein plus gel (36270ES10, Yeasen) and subsequently transferred to PVDF membranes. The membranes were blocked in 5% silk milk for 80 min and then incubated with primary antibodies overnight at 4 °C. Secondary antibodies were employed the following day. The protein bands were visualized using the Chemiluminescence Imaging System (Bio‐Rad, USA), and the gray values were analyzed using ImageJ software.

### qRT‐PCR Analysis

mRNA expressions of FOXC2, Fibronectin, α‐SMA, E‐cadherin, collagen I, TGF‐β1, Smad2, and Smad3 were assessed by qRT‐PCR, with GAPDH serving as the internal control. Total RNA was extracted from cecum tissue and cell samples, then synthesized, quantified, and amplified, following the instructions. The primers were designed by Tsingke Co., Ltd. (Beijing), and the primer pair sequences were outlined in Supplementary Table S1. The relative transcription levels of the target genes were determined using the comparative Ct (2^−ΔΔCt^) method.

### Statistical Analysis

The data were analyzed using SPSS 25.0 software (Chicago, USA) and were presented as mean ± standard deviation. For normally distributed continuous data, a One‐way ANOVA test with a Tukey's test was employed, while the Kruskal–Wallis test was utilized for data that did not follow a normal distribution. A *p*‐value of < 0.05 was considered statistically significant.

## Conflict of Interest

The authors declare no conflict of interest.

## Author Contributions

L.Y. and Y.F. contributed equally to this work. L.Y. and Y.B. designed and supervised the study. Y.F., Y.L., Z.K., J.M., Y.C., W.L., F.C., and Y.C. performed the most experiments. B.Z. analyzed the results. L.Y. and Y.F. drafted the manuscript. L.Y. and Y.B. critically revised drafts of the manuscript. All authors reviewed the manuscript.

## Supporting information



Supporting Information

## Data Availability

The data that support the findings of this study are available from the corresponding author upon reasonable request.
