# Supplementary material for: Aloe‐Emodin Targeting FOXC2 Disrupts NETs Formation and EMT‐Driven Postoperative Peritoneal Adhesion Through TGF‐β1‐Smad2/3 Pathway
Source: Adv Sci (Weinh). 2025 Oct 13;12(48):e11013. doi: 10.1002/advs.202511013 (PMC12752568; doi:10.1002/advs.202511013)
Supplement: Supplementary file 1 — Supporting Information [file ADVS-12-e11013-s001.docx]

**Aloe-emodin Targeting FOXC2 Disrupts NETs Formation and EMT-Driven Postoperative Peritoneal Adhesion through** **TGF-β1-Smad2/3 pathway**

Lili Yang^1,2,ǂ^, Yunda Fang^3,ǂ^, Yuheng Lian^4^, Ziyang Kong^5^, Jia Miao^3^, Yanqi Chen^3^, Wen Li^5^, Feiyan Chen^6^, Bin Zhang^7^, Yao Chen^8^, Yaoyao Bian^5,9,*^

*^1^ Jiangsu Provincial Engineering Research Center of TCM External Medication Development and Application, Nanjing University of Chinese Medicine, Nanjing, 210023, China*

*^2^ Jingwen Library, Nanjing University of Chinese Medicine, Nanjing, 210023, China*

*^3^* *School of First Clinical Medicine, Nanjing University of Chinese Medicine, Nanjing 210023, China*

*^4^ Faculty of Chinese Medicine, Macau University of Science and Technology, Taipa, Macau 999078, China*

*^5^ School of Health Preservation and Rehabilitation, Nanjing University of Chinese Medicine, Nanjing, 210023, China*

*^6^ Research and Innovation Center, College of Traditional Chinese Medicine, Integrated Chinese and Western Medicine College, Nanjing University of Chinese Medicine, Nanjing 210023, China.*

*^7^Department of Gastroenterology, Ningbo Municipal Hospital of TCM, Affiliated Hospital of Zhejiang Chinese Medical University, Ningbo, 315012, China.*

*^8^ School of Pharmacy, Nanjing University of Chinese Medicine, Nanjing 210023, China*

*^9^ TCM Rehabilitation Center, Jiangsu Second Chinese Medicine Hospital, Nanjing 210023, China*

^ǂ^ LLY and YDF contributed equally to this work.

^*^ Yaoyao Bian − Ph.D., Professor, School of Health Preservation and Rehabilitation, Nanjing University of Chinese Medicine, Nanjing, Jiangsu 210023; E-mail: [bian@njucm.edu.cn](mailto:bian@njucm.edu.cn).

Table S1. The primer used for qRT-PCR

| Species | Genes | Primer sequence (5’-3’) |
| --- | --- | --- |
| Ratus norvegicus | FOXC2-F | GCCCAGCAGCAAACTTTCC |
|  | FOXC2-R | CCCGAGGGTCGAGTTCTCA |
|  | Fibronectin-F | CCTTAAGCCTTCTGCTCTGG |
|  | Fibronectin-R | CGGCAAAAGAAAGCAGAACT |
|  | αSMA-F | CATCAGGAACCTCGAGAAGC |
|  | αSMA-R | TCGGATACTTCAGGGTCAGG |
|  | E-cadherin-F | CAGGATTACAAGTTCCCGCCA |
|  | E-cadherin-R | CACTGTCCGCTGCCTTCA |
|  | collagen Ⅰ-F | GGCATAAAGGGTCATCGTG |
|  | collagen Ⅰ-R | GAACCTTCGCTTCCATACTC |
|  | TGF-β1-F | GAGAGCCCTGGATACCAACTACTGC |
|  | TGF-β1-R | CAACCCAGGTCCTTCCTAAAGTCAA |
|  | Smad2-F | GCCGAGTGCCTAAGTGAT |
|  | Smad2-R | AGACTGAGCCAGAAGAGC |
|  | Smad3-F | TGTCATCTACTGCCGCTTGTG |
|  | Smad3-R | CAACACTGGAGGTAGCACTGG |
|  | GAPDH-F | CATGCCGCCTGGAGAAACC |
|  | GAPDH-R | GCCAGCCCCAGCATCAAAG |
| Homo sapiens | FOXC2-F | ATGCAGGCGCGCTACTCCGTGTCCGACCCC |
|  | FOXC2-R | GTATTTCGTGCAGTCGTAGGAGTAGGGGGCTG |
|  | Fibronectin-F | CCATCGCAAACCGCTGCCAT |
|  | Fibronectin-R | AACACTTCTCAGCTATGGGCTT |
|  | αSMA-F | GCGTGGCTATTCCTTGGTTA |
|  | αSMA-R | TGATGCTGTTGTAGGTGGTTTC |
|  | E-cadherin-F | TCGACACCCGATTCAAAGTGG |
|  | E-cadherin-R | TTCCAGAAACGGAGGCCTGAT |
|  | GAPDH-F | GGGGCTCTCCAGAACATCATCC |
|  | GAPDH-R | ACGCCTGCTTCACCACCTTCTT |
